# Exploring general-purpose protein features for distinguishing enzymes and non-enzymes within *the twilight zone*

**DOI:** 10.1186/s12859-017-1758-x

**Published:** 2017-07-21

**Authors:** Yasser B. Ruiz-Blanco, Guillermin Agüero-Chapin, Enrique García-Hernández, Orlando Álvarez, Agostinho Antunes, James Green

**Affiliations:** 1 Facultad de Química y Farmacia, Universidad Central “Marta Abreu” de Las Villas, 54830 Santa Clara, Cuba; 2 CIMAR/CIIMAR, Centro Interdisciplinar de Investigação Marinha e Ambiental, Universidade do Porto, Terminal de Cruzeiros do Porto de Leixões, Av. General Norton de Matos, s/n, 4450-208 Porto, Portugal; 3 Centro de Bioactivos Químicos (CBQ), Universidad Central ¨Marta Abreu¨ de Las Villas (UCLV), 54830 Santa Clara, Cuba; 4 Instituto de Química, Universidad Nacional Autónoma de México (UNAM), 04360 D.F, México, Mexico; 5 Departamento de Biologia, Faculdade de Ciências, Universidade do Porto, Rua do Campo Alegre, 4169-007 Porto, Portugal; 6 Department of Systems and Computer Engineering, Carleton University, Ottawa, Canada; 7 Theoretical Chemistry, Max Planck Institute für Kohlenforschung, 45470 Mulheim an der Ruhr, Germany

**Keywords:** Enzyme, Alignment-free protein analysis, Protein descriptors, Support vector machines, ProtDCal, TI2BioP

## Abstract

**Background:**

Computational prediction of protein function constitutes one of the more complex problems in Bioinformatics, because of the diversity of functions and mechanisms in that proteins exert in nature. This issue is reinforced especially for proteins that share very low primary or tertiary structure similarity to existing annotated proteomes. In this sense, new alignment-free (AF) tools are needed to overcome the inherent limitations of classic alignment-based approaches to this issue. We have recently introduced AF protein-numerical-encoding programs (TI2BioP and ProtDCal), whose sequence-based features have been successfully applied to detect remote protein homologs, post-translational modifications and antibacterial peptides. Here we aim to demonstrate the applicability of 4 AF protein descriptor families, implemented in our programs, for the identification enzyme-like proteins. At the same time, the use of our novel family of 3D–structure-based descriptors is introduced for the first time. The Dobson & Doig (D&D) benchmark dataset is used for the evaluation of our AF protein descriptors, because of its proven structural diversity that permits one to emulate an experiment within the *twilight zone* of alignment-based methods (pair-wise identity <30%). The performance of our sequence-based predictor was further assessed using a subset of formerly uncharacterized proteins which currently represent a benchmark annotation dataset.

**Results:**

Four protein descriptor families (sequence-composition-based (0D), linear-topology-based (1D), pseudo-fold-topology-based (2D) and 3D–structure features (3D), were assessed using the D&D benchmark dataset. We show that only the families of ProtDCal’s descriptors (0D, 1D and 3D) encode significant information for enzymes and non-enzymes discrimination. The obtained 3D–structure-based classifier ranked first among several other SVM-based methods assessed in this dataset. Furthermore, the model leveraging 1D descriptors, showed a higher success rate than EzyPred on a benchmark annotation dataset from the *Shewanella oneidensis* proteome.

**Conclusions:**

The applicability of ProtDCal as a general-purpose-AF protein modelling method is illustrated through the discrimination between two comprehensive protein functional classes. The observed performances using the highly diverse D&D dataset, and the set of formerly uncharacterized (hard-to-annotate) proteins of *Shewanella oneidensis*, places our methodology on the top range of methods to model and predict protein function using alignment-free approaches.

**Electronic supplementary material:**

The online version of this article (doi:10.1186/s12859-017-1758-x) contains supplementary material, which is available to authorized users.

## Background

Advances in both next-generation sequencing (NGS) technologies and mass spectrometry-based proteomics have allowed the continuous growth of available proteomes and metaproteomes in biological databases. However, the high protein structural variety in known proteomes makes the protein functional characterization a challenging task in modern Computational Biology and Bioinformatics [1]. As manually curated annotations are available only for a small portion of investigated systems; the wealth of genomic and transcriptomic information generated from NGS technologies [2] requires the use of accurate computational annotation tools [3]. The same is true for the functional annotation of 3D structures in databases such as the PDB [4], SCOP [5] and CATH [6], as biologically uncharacterized proteins are being incorporated continuously in these databases; currently about 3725 structures in the PDB have a classification of ‘unknown function’.

The assignment of a functional class for a query protein is a complex problem, not just because of the structural complexity but, because a single protein can have multiple functions, either due to its multiple domains or its subcellular locations and substrate concentrations [7]. Nevertheless, protein functional inferences have traditionally relied on structural/sequence similarities provided by alignment-based algorithms. The most common alignment-based (AB) approaches used in genomic and amino acid sequence databases to identify protein functional signals include: the Smith Waterman algorithm [8], the Basic Local Alignment Search Tool (BLAST) suite of programs [9], and profile Hidden Markov Models (HMMs) [10]. Profile HMM are at the core of the popular Protein family (Pfam) database [11]. Particularly for an effective identification of enzymatic functions within proteomes, BLAST and HMMs have been implemented in the annotation pipeline of EnzymeDetector along with the integration of the main biological databases [12].

Despite the large success of these methods, sequence-similarity-based approaches often fail when attempting to align proteins that share less than 30–40% identity. Alignments within this so-called *twilight zone* are often unreliable, resulting in reduced prediction accuracy [13, 14]. This handicap has caused a sustained increase in the number of unannotated proteins during the examination of genomes and proteomes from a variety of organism and environmental samples. Consequently, alignment-free (AF) approaches are needed to overcome such limitations, to accurately detect gene/protein signatures within the twilight zone, and to provide clues about the functional classes e.g. enzymes or non-enzymes for subsets of uncharacterized proteins.

Given the supremacy of AB approaches for predicting the function of a protein, we considered interesting and valuable to dig into the state of the art of AF methods and make our own contribution in this field. In this sense, we believe that the development of general-purposes AF prediction methods, based on new protein structure descriptors, can contribute to enhance the predictability of protein functional classes such as those of top hierarchy: enzymes and non-enzymes. This discrimination challenges current classification approaches due to their intrinsic structural and functional diversity.

Generally, AF methods have been based on amino acid composition description, such as the one reported in Ref. [15] to detect remote members of the of G-protein-coupled receptor superfamily using support vector machines (SVMs). Also, AF descriptors such as the amino acid content and the amino-acid-pair-association rules, were used along with several classification methods to categorize protein sequences [16]. The web-server Composition-based Protein identification (COPid) was developed to annotate the function of a full or partial protein strictly from its composition [17].

One of the most popular AF protein features are those based on Chou’s concept of pseudo amino acid composition (PseAAC), initially used to leverage the effect of sequence order together with the amino acid composition for improving the prediction quality of protein cellular attributes [18]. This concept has been widely used to predict many protein attributes [19–21] including functional assignments such as whether a protein sequence is an enzyme or not, as well as the enzyme class they belong to [22, 23]. The experience achieved by Chou et al. in detecting and sub-classifying enzyme-like proteins was summarized in the EzyPred webserver [24].

In a similar way to the Chou’s descriptors, Caballero and Fernandez defined Amino Acid Sequence Autocorrelation (AASA) vectors, but, instead of using a distance function (difference between pairs of a property values) like in the PseAAC, they used autocorrelation (multiplication of a property values). This latter approach was applied to predict the conformational stability of human lysozyme mutants [25]. AASA is an extension of the Broto-Moreau autocorrelation topological indices previously used in structure-activity relationship (SAR) studies of protein sequences [26]. Until recently, the most comprehensive computational tool for the generation of AF descriptors of amino acid sequences was the server PROFEAT [27]. This server gathers most of the above-mentioned approaches in a flexible computational tool enabling the generation of thousands of features per query protein.

Other efforts for efficient numerical encoding of proteins involve the extension of molecular descriptors, originally defined for small and mid-sized molecules, into protein descriptors. Following this methodology, Gonzalez-Diaz et al. have extended their Markovian stochastic descriptors to characterize protein sequences [28]. In addition, graphical approaches have been validated and implemented in our program TI2BioP (Topological Indices to BioPolymers), which allows the calculation of spectral moments as topological indices from different 2D graphical approaches for DNA, RNA, and protein biopolymers [29].

We have recently introduced ProtDCal, a software package for the general-purpose-numeric encoding of both protein sequences and structures [30]. This software uses a distinctive *divide-and-conquer* methodology based on extracting diverse groups of amino acids and aggregating the contributions of the residues in each group into scalar descriptors, giving rise to a vast number of features that balance local and global characteristics of the protein sequence and structure. Principal component analysis has been used to demonstrate the distinct information content of ProtDCal’s descriptors relative to PROFEAT among representatives from the different sequence-based descriptor families encoded by these two programs. The applicability of ProtDCal’s sequence-based descriptors for automatic functional annotation was first illustrated in the classification of the N-glycosylation state of asparagine residues of human and mammalian proteins [30, 31]. Recently, sequence-based features derived from ProtDCal were also used in the development of a multi-target predictor of antibacterial peptides against 50 Gram positive bacteria [32]. However, the utility of the 3D structure features generated using ProtDCal still have not been demonstrated. Therefore, firstly, this work aims to validate the applicability of different families of descriptors implemented in TI2BioP and ProtDCal for the discrimination between enzymes and non-enzymes using the structurally non-redundant benchmark dataset designed by Dobson and Doig (D&D) [33]. In a second step, the obtained model is applied to distinguish enzymes and non-enzymes among a subset of uncharacterized proteins.

The descriptors of our programs represent the four largest families of AF descriptors: sequence-composition-based (0D), linear-topology-based (1D), pseudo-fold-topology-based (2D) and 3D–structure features (3D). The 0D, 1D and 3D protein descriptor families are calculated by means of ProtDCal while the 2D descriptors are generated by TI2BioP. More information about the descriptor classes can be found in Additional file 1.

We show the superior performance of a model using 3D information represented by ProtDCal’s features, relative to the previously developed 3D methods. In addition, we introduce a model using sequence-based features that rivals several of the 3D–structure-based methods evaluated on the same data. This model was comparatively evaluated with Ezypred and EnzymeDetector on 30 proteins which were originally uncharacterized during the annotation of the *Shewanella oneidensis* proteome in 2002, and currently represent a benchmark annotation dataset [34]. Our model achieves a higher success rate than EzyPred. Such a result highlights that our general-purpose protein descriptors, followed by supervised feature selection, can efficiently encode subtle structural elements that distinguish enzymes from non-enzyme proteins.

## Methods

### Dataset

The described SVM-based models were trained and cross-validated using the D&D benchmark dataset, which consists of 1178 structurally diverse proteins, comprising 691 enzymes and 487 non-enzymes, based on annotations in the PDB and Medline abstracts. The same external dataset of 52 proteins, used by Dobson and Doig to assess their model, is also used in the present report as an external test for performance comparison [33].

### Generation of AF protein features

#### ProtDCal protein features

**Fig. 1:**
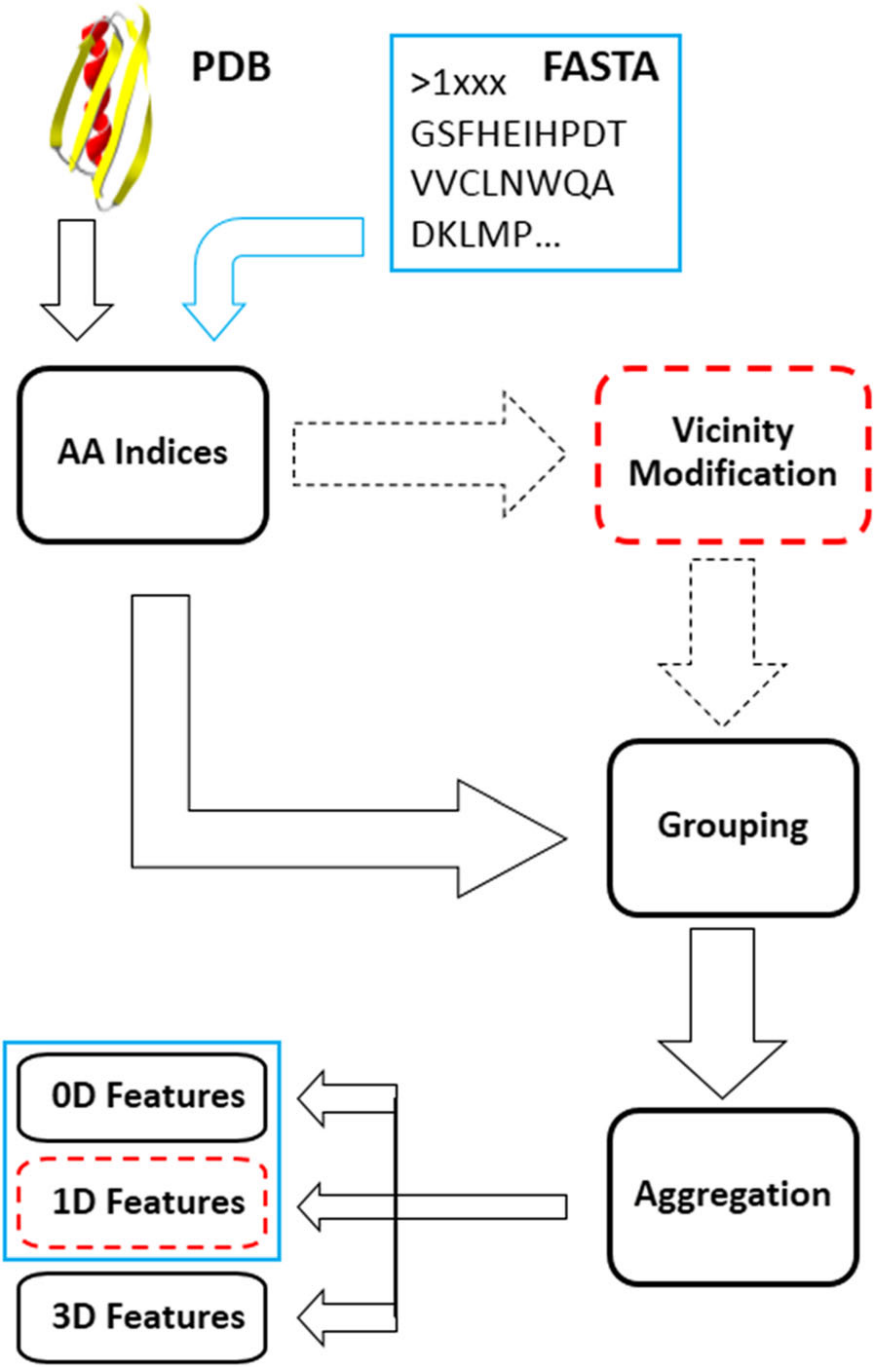
Schematic representation of the protein descriptor generation process of ProtDCal. The dashed drawings denote an alternative pathway in the feature generation, which leads to a different family of descriptors. The *blue* drawings indicate those families of descriptors derived purely from primary structure information

Figure 1 depicts the process followed in ProtDCal to obtain the final features. Either sequences in FASTA format or structures in PDB files can be used as input for the program. Individual descriptors arise from the combinatorial mixing of different property values for the 20 regular residues, which are subsequently modified according to their neighbours, and then grouped by types. Lastly, the modified contributions within every group are aggregated with an invariant operator to create a scalar numeric quantity.

Below we describe each of these steps in more detail, although an exhaustive description can be found in our paper introducing ProtDCal [30] and in the documentation of the program. In a recent report, a similar features generation setup was employed, but only using sequence-based descriptors [31].

Step 1: Numeric codification of residues. The numerical value of an amino acid property is used to build an initial array associated to each residue in a protein. Several properties can be used, giving rise to the same number of individual arrays. ProtDCal implements different indices used to primarily encode the residues in order to compute sequence-based (0D, 1D) protein features. These indices comprise diverse structural and chemical-physical properties of amino acids taken, mostly, from the AAindex database [35]. Each type of amino acid index can be selected for the codification of the residues, giving rise to a corresponding array of values representing all the protein. The summary of the sequence-based indices is presented in Additional file 2: Tables SI-1 and SI-2.

In the present study, the calculation of sequence-based features was conducted using 16 amino acid indices: 1–3) The so-called principal properties or z-values (z1, z2 and z3) [36], which are associated with hydrophilicity, steric, and electronic properties of each type of amino acid, respectively; 4) The molecular mass of amino acids (Mw); 5–7) The three Levitt’s probabilities to adopt α-helix (pa), β-sheet (pb) or β-turn (pt) conformations [37]; 8) The isoelectric point (IP); 9) The superficial free energy (ΔGs(U)), defined as the product of the hydrophobicity according to Kyte&Doolitle’s scale [38] and total surface area of the isolated amino acid; 10) The polar area (Ap); 11) The hydrophobicity according Kyte&Doolitle’s scale [38]; 12) The Electronic Charge Index (ECI) [39]; 13) The Isotropic Surface Area (ISA) [39]; 14) The enthalpy of formation of a nonapeptide centered on the given residue and flanked with +/− 4 ALA residues (ΔHf) [40]; and 15–16) The compatibility parameters L1–9 and Xi introduced by [40]. Most of these AA properties appear in the AAindex database [35] and a more detailed description of each can also be found in ProtDCal’s documentation.

In order to generate 3D descriptors, structural-amino-acid indices are used to encode each residue in a protein. Here, 29 indices were calculated, comprising: 1–8) Eight indices associated with the dihedral angles, phi and psi, of the protein’s backbone (wPsiH, wPsiS, wPsiI, wPhiH, wPhiS, wPhiI, Phi, Psi); 9–10) The accessible surface area (A) and the superficiality index (wSp); 11) The buried non-polar area (ΔAnp); 12) A measure of the folding degree (lnFD) introduced in our previous report [41]; 13) The squared radius of the protein (wR2); 14–20) Seven contact-based indices (wNc, wFLC, wNLC, wCO, wLCO, wRWCO, wCTP), each one weighted with seven of the above mentioned amino acid properties (HP, ECI, IP, Z1, Z2, Z3, ISA), in order to distinguish contacts involving different residues; 21–29) Nine thermodynamic indices (Gw(F), Gs(F), W(F), ΔGs, HBd, ΔGel, ΔGw, ΔGLJ, ΔGtor) associated with the number of hydrogen bonds in the backbone of the protein and several empirical approaches capturing folding free energy contributions [42, 43] referring to Lenard-Jones and electrostatic interactions, torsion potential, superficial free energy, hydrophobic effect, etc. A summary of all the structure-based indices is presented in the Additional file 2: Tables SI-3 and SI-4.

Step 2: Modification by vicinity. Once these arrays of indices are formed, their numeric values are altered according to the values of the neighbouring residues. Several vicinity operators are associated with different definitions of neighbourhood. In the present work, we use the Electro-topological State (ES), where the vicinity of each residue is defined by all the other residues in the protein. The influence of each neighbour residue in the ES operator is determined by the sequence separation between the pairs of residues. Other operators, like the Autocorrelation (AC), considers a restricted vicinity comprising only those residues at specific sequence separation from the central position that is being modified. As a rule of thumb, we encourage the use of a global vicinity operator like ES when modelling global properties as is the case of this work, i.e. those that reflect the protein as a whole and not to local sites as might be appropriate when trying to predict post-translational modifications. The modification process is applied independently for each initial individual array. The family of 0D features is obtained directly from the original set of values from Step 1, without applying any vicinity operator at this stage. Using a vicinity-modification operator over the values of a given index for all the residues, permits one to incorporate information about the order of the amino acids into the resulting descriptor value. Thus, the application of these operators is the key step to transforming the 0D residue indices into the final 1D descriptors (see red-dashed squares in Fig. 1).

In the present study, 1D descriptors were obtaining by applying the Electrotopological State (ES) operator. The ES, originally defined by Kier and Hall [44, 45], describes the information related to the electronic and topological state of the atom in the molecule as:

$$ {ES}_i={I}_i+\Delta {I}_i={I}_i+\sum_{j=1}^N\frac{I_i-{I}_j}{{\left({d}_{i j}+1\right)}^2} $$

Where I_i_ is the intrinsic state of the i^th^ atom and ΔI_i_ is the field effect on the i^th^ atom representing the perturbation of the intrinsic state of the i^th^ atom by all other atoms in the molecule. The remaining terms are d_ij_, the topological distance between the i^th^ and the j^th^ atoms, and N, the total number of atoms. The intrinsic state is defined as a quantity that relates the principal quantum number, the number of valence electrons, and the number of bonds or sigma electrons of the atom. When applying this operator to proteins, one considers the sequence of residues as the topological nodes of a linear molecular graph. The intrinsic state of a given residue is taken as the value of a selected amino acid index (from Step 1). The topological distance is computed as the number of residues between the i^th^ and the j^th^ amino acids (d_ij_ = |j – i|).

Step 3: Grouping. This stage splits each array of modified index values of the protein into a set of subarrays associated to groups of residues (not necessarily connected). Many grouping criteria are implemented in ProtDCal allowing one to form subarrays containing the altered index values for each selected residue within the group. The groups can vary both in size and composition; on one hand the largest group is formed by the entire protein and, on the other hand, the most specific groups can gather only a single type of residue or even a single residue position in the chain. There are more flexible groups that specify residue types such as all hydrophobic, aromatic, or polar residues. Such partitioning of the information contained in an amino-acid sequence allows obtaining features with high concentration of relevant information for a given problem. Such relevant features should be identified by means of supervised attributes selection processes in subsequent steps of the modelling. The grouping process is applied independently for each modified array. Here, 32 groups of residues were extracted as follows: 1–20) the 20 natural residue types (alanine, arginine, tyrosine, etc.); 21–29) nine groups formed according to physical and structural properties of the amino acids (hydrophilic, non-polar, aromatics, etc.); 30) the entire protein is taken as a special group including all the residues; 31–32) two groups comprising the internal and the superficial residues were created exclusively for the calculation of 3D descriptors. See Additional file 2: Table SI-5 for a complete list and description of the groups.

Step 4: Invariant aggregation. Every subarray of modified indices, formed in the previous step, is transformed into a single scalar value through an aggregation operator. Many of such aggregation operators are implemented in ProtDCal, where the simplest is the sum of all the elements of the subarray. Such operators are organized in the program by category, such as norms, central tendency, dispersion and information theoretic measures. Each of these types of formalisms characterize aspects of the structural information in each group of residues that leads to another level of segregation of the original information in the protein. The aggregation operators are created by the p-norms of orders *p* = 1 to *p* = 3 [46], central-tendency measures (average, geometric and harmonic means, etc.), dispersion and distribution parameters (variance, kurtosis, skewness, quartiles, etc.) and information-theoretic measures based on Shannon entropy [47]. This final step transforms the set of values associated with a given group of residues into a single value that represents the final descriptor. A total of 17 such operators was used to obtain the final sets of features for the 0D, 1D and 3D descriptor families (see Additional file 2: Tables SI-6 to SI-9).

The different indices, groups, and operators selected through these four stages are combined to generate a large set of features for each protein. The descriptors are labelled using the format: <Index>_ < Mod. Op. > _ < Group>_ < Aggr. Op.>. For instance, the descriptor HP_NO_ARM_Ar corresponds to the average (Ar) of the hydrophobicity (HP) values for all the aromatic (ARM) residues in the protein. The tag NO indicates that no vicinity operator was applied (thereby producing a 0D descriptor). The descriptor HP_ES_ARM_Ar corresponds to the 1D type because the Electrotopological State (ES) is used to modify the hydrophobicity values of each residue according the sequence separation to its neighbours. The feature wCTP(IP)_NO_PHE_N2 is a 3D descriptor, since it uses the 3D structure to compute the Chain Topology Parameter (CTP) [48] to encode all the phenylalanine residues (PHE), which spatial contacts are in turn weighted with the product of the isoelectric points (IP) of the residues forming the contacts. No vicinity operator is applied in this case, and the p-norm with *p* = 2 is used as the aggregation operator for this descriptor.

#### TI2BioP pseudo-folding (2D) features

**Fig. 2:**
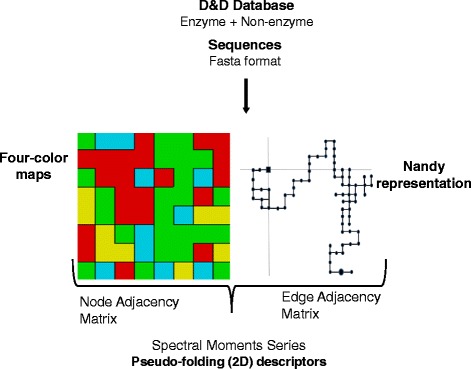
Workflow for the calculation of the pseudo-fold 2D indices (spectral moments series) in TI2BioP. Illustrations of both, the Nandy and FCM representations of a graph are presented

TI2BioP (Topological Indices to BioPolymers) projects long biopolymeric sequences into 2D artificial graphs, such as Cartesian (Nandy) and four-color maps (FCMs), but also reads other 2D graphs from the thermodynamic folding of DNA/RNA strings inferred from other programs. The topology of such 2D graphs is either encoded by node or adjacency matrices for the calculation of the spectral moments (μ), thus obtaining pseudo-fold 2D descriptors. In this study, spectral moment series (μ_0_ - μ_15_) were computed using FCMs and Nandy’s representation (Fig. 2).

A total of 56 amino acid properties were used to weight the contributions of each residue to the spectral moment’s estimation. Spectral moments series (from 0th to 15th order) are calculated either considering the influence over a certain node or edge (i) of the graph of other nodes/edges (j) placed at different topological distances (0–15) determined by their coordinates in the artificial 2D graph. Notice that each node represents a cluster of amino acids showing similar physico-chemical properties and the edge connecting both nodes is weighted by the average of the properties between two bound nodes. For further information about the calculation of these indices, please refer to the following references [29, 49].

### Feature selection strategy

#### Information gain (IG) filtering

Information entropy, originally proposed by Shannon, is considered to be the most important concept in information theory. Shannon entropy is the expected value of the uncertainty for a given random variable. High uncertainty can correspond to more information, therefore, entropy provides a quantitative measure of information content [50]. IG measures the loss of information entropy when a given variable is used to group values of another variable. It can thus be considered a measure of the degree of information ordering of an outcome variable when using an independent variable to reproduce the distribution of the outcome [51]. Several information-theoretic-based approaches have been proposed for feature selection [52–54]. Here, IG is used as a feature selection method to distinguish the descriptors that most influence the discrimination between enzyme and non-enzyme proteins. IG is formulated as the difference between the Shannon entropy of a variable X and the conditional entropy of X given a second variable Y:

$$ {IG}_c\left( X| Y\right)= H(X)-{H}_c\left( X| Y\right) $$

where X is the class variable (i.e., enzyme and non-enzyme proteins). The first term represents the total information needed to describe the class distribution of the data set used. While the conditional term represents the missing information needed to describe the class variable knowing the descriptor Y. The formulations for each of these terms are:

$$ H(X)=-\sum_i P\left({x}_i\right){ \log}_2\left( P\left({x}_i\right)\right)\kern1em  i=1,2 $$

$$ {H}_c\left( X| Y\right)=-\sum_j{P}_c\left({y}_j\right)\sum_i{P}_c\left({x}_i|{y}_j\right){ \log}_2\left({P}_c\left({x}_i|{y}_j\right)\right) $$

where P(x) is the prior probability of each class, calculated as the fraction of the number of instances of class X in the total number of instances in the dataset; P_c_(x|y) is the conditional probability of the X class given certain values of descriptor Y, which is obtained as the fraction of instances within class X among a set of cases selected according to the values of the descriptor Y; and P_c_(y) represents the probability of a subset of cases, selected according to their values of Y. This latter probability is obtained as the ratio between the number of cases in the subset and the number of cases in the dataset. P_c_(y) allows obtaining a weighted average of the conditional entropy of different subsets, defined by the values of descriptor Y, resulting in the conditional entropy of the class variable X given a descriptor Y.

#### Redundancy reduction

A single-linkage clustering strategy was implemented using the Spearman correlation coefficient (ρ) as a measure of pairwise similarity among the features. Once the clusters of features are built, the closest descriptor to the centroid of each cluster is identified and extracted to create the subset that is analysed in the next step of the features selection. This algorithm is implemented in a Perl script that can be found within the ‘Utils’ directory of the ProtDCal distribution, guidelines of how to use it are described within the file.

#### Supervised selection of the best subsets

The final best subset of features is extracted by assessing the performances in cross-validation (CV) of SVM models trained with subsets of features extracted along a Genetic Search [55] over the feature space.

The detailed feature selection pipeline is as follow: first, the program Weka [56] is used to rank the features according to their Information Gain (IG). Only those features with IG values representing 15% of the total information content of the class distribution are extracted for further analyses. Then, a single-linkage clustering is performed, with a Spearman correlation cutoff of ρ = 0.95 to link two neighbors in a cluster. The closest element to the centroids of each cluster are extracted as representative. Next, we use the WrapperSubsetEval method implemented in Weka (version 3.7.11 or higher) to search for an optimum subset of features. The wrapper class is used with the GeneticSearch method and each trial subset is scored according to the F1-measure for the positive class obtained in a 5-fold cross-validation test with an SVM classifier trained with Weka’s default set of parameters. Table 1 summarizes the number of features remaining after each selection step, for every class of descriptor.

**Table 1:** Number of remaining features for each one of the protein descriptor families after applying several selection filters

Set	Initial	Info. Gain	Redundancy	Best Subset
0D	3905	891	34	9
1D	8705	1456	265	13
2D	1883	1256	5	5
3D	64,313	8339	2456	26

### SVM-based models building

SVM-based models were obtained and validated with a scheme of 10 × 10-fold CV using random splits of the data according to the implementation of the CV test in Weka. Ten CV runs were conducted by changing the seed of the random number generator in order to automatically generate different splits of the dataset for each run. The average performance of the 10 CV runs is reported, together with the standard deviation of this performance. Such deviation represents an estimation of the error of the predicted accuracy because of variations in training and validation data.

## Results and discussion

### D&D: A benchmarking dataset for alignment-free approaches

D&D designed a benchmark dataset by applying 3D–structural constraints in order to ensure a large structural diversity and representativeness in the data [33], despite the wide use of this data for assessing 3D–structure-based classification methods, this dataset has not been carefully examined by sequence similarity analyses, which is necessary to assure the transferability of the attained performances during the assessment of AF methods.

For many years, pairwise sequence identity was the most common similarity measure to define the named twilight zone for alignment-based algorithms (<30% of amino acid identity). Sequence alignments frequently fail to identify homology within this similarity zone [13]. However, more recently, it has been recognized that the “30% of identity” rule of thumb underestimates the number of homologs that can be detected by sequence similarity. In this sense, the bit score and its associated e-value have been shown to be better measures for detecting homology [7]. According to Pearson (2003), for average length proteins, a bit score of 40 is significant (E < 0.001) in searches of protein databases with fewer than 7000 entries [7].

In this sense, we here evaluate the sequence similarity within the D&D dataset by using two similarity measures: the percent of identities from global (Needleman- Wunsch) and local (Smith-Waterman) alignments, as well as the bit scores from BLAST.

**Fig. 3:**
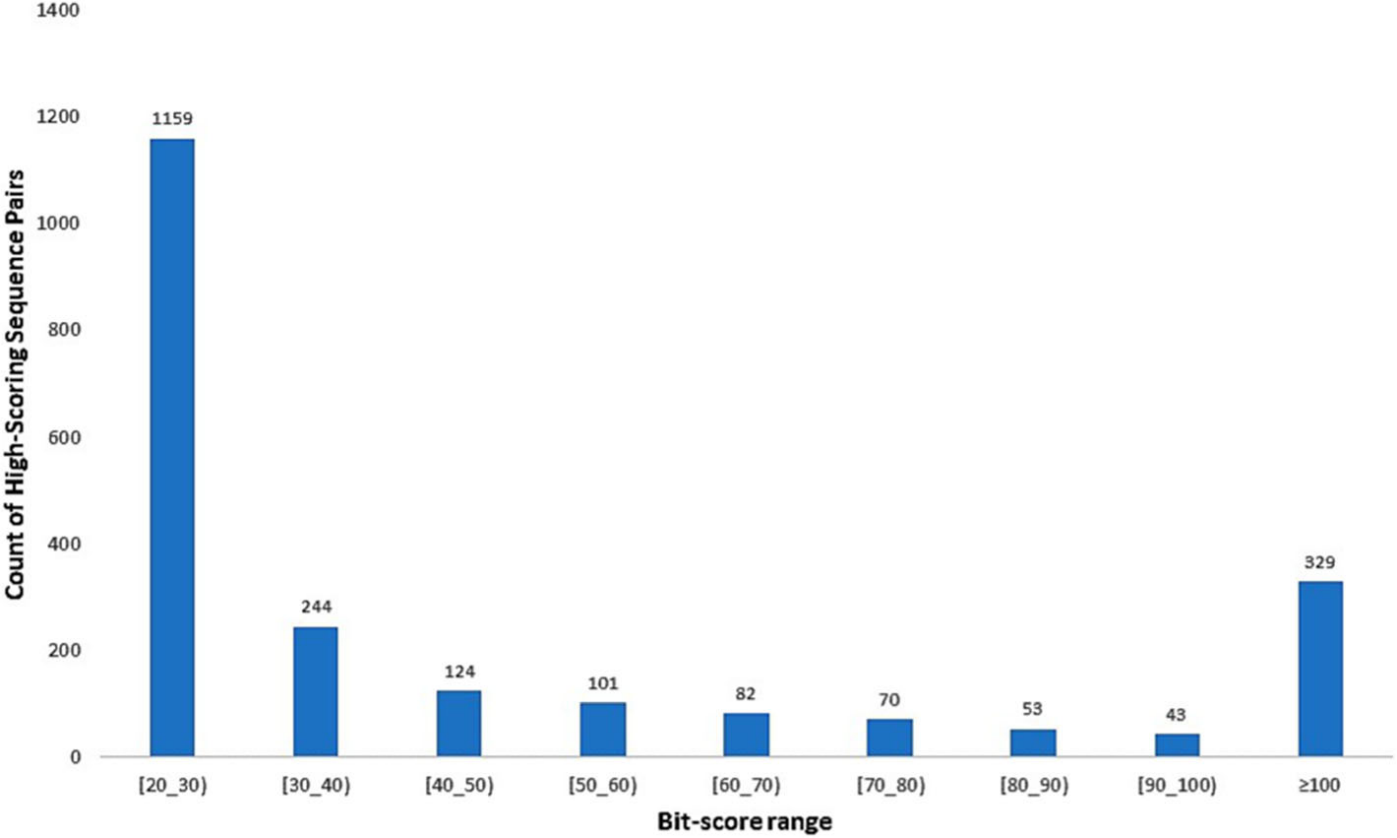
Distribution of the number of High-scoring Sequence Pairs according to Bit-Score value ranges. Each sequence pair is represented by the highest scoring segment pair (HSP) in the local alignment. HSP were obtained with BLAST using a permissive e-value cutoff = 10

The dot plot resulting from the global and local *all-*vs*-all* sequence alignments showed an overall blue landscape evidencing the low degree of global and local identity among the sequences in the dataset (Additional file 2: Figure SI-1). Most protein pairs in D&D dataset share less than 30% of amino acid identity, confirming that is a structurally non-redundant subset from PDB. The analysis of the bit-scores associated to the high-scoring segments pairs (HSPs) (bit score > 40) between pairs of sequences, highlighted a very small fraction of biologically related sequence pairs (putative homologs), representing 802 pairs out of the 693,253 possible sequence pairs in the dataset (Fig. 3). Additionally, only 2205 (0.3%) out of the total pairs showed at least one HSP with an e-value lower than the used cut-off of 10. These results illustrate the low overall similarity present within the D&D dataset.

On the other hand, we additionally explored the structural diversity among the enzyme and non-enzymes subsets according to SCOP’s hierarchical structural levels [57]. Both classes are distributed among all the root structural classes (all-α, all-β, α/β, α + β, multi-domain, etc.). They were also subsequently distributed among several folds and superfamilies within each class (see Additional file 2: Figure SI-2, Tables SI-10 and SI-11). We conclude that the D&D is, on average, a highly diverse and representative dataset, which is suitable for the evaluation of both 3D structure-based methods and alignment-free sequence-based predictors.

### Description of extracted subsets of AF features

The different families of AF features were screened through the three following filtering stages described in Methods section: *Information Gain (IG) filtering, Redundancy reduction and Supervised selection of the best subsets* ***.***

**Fig. 4:**
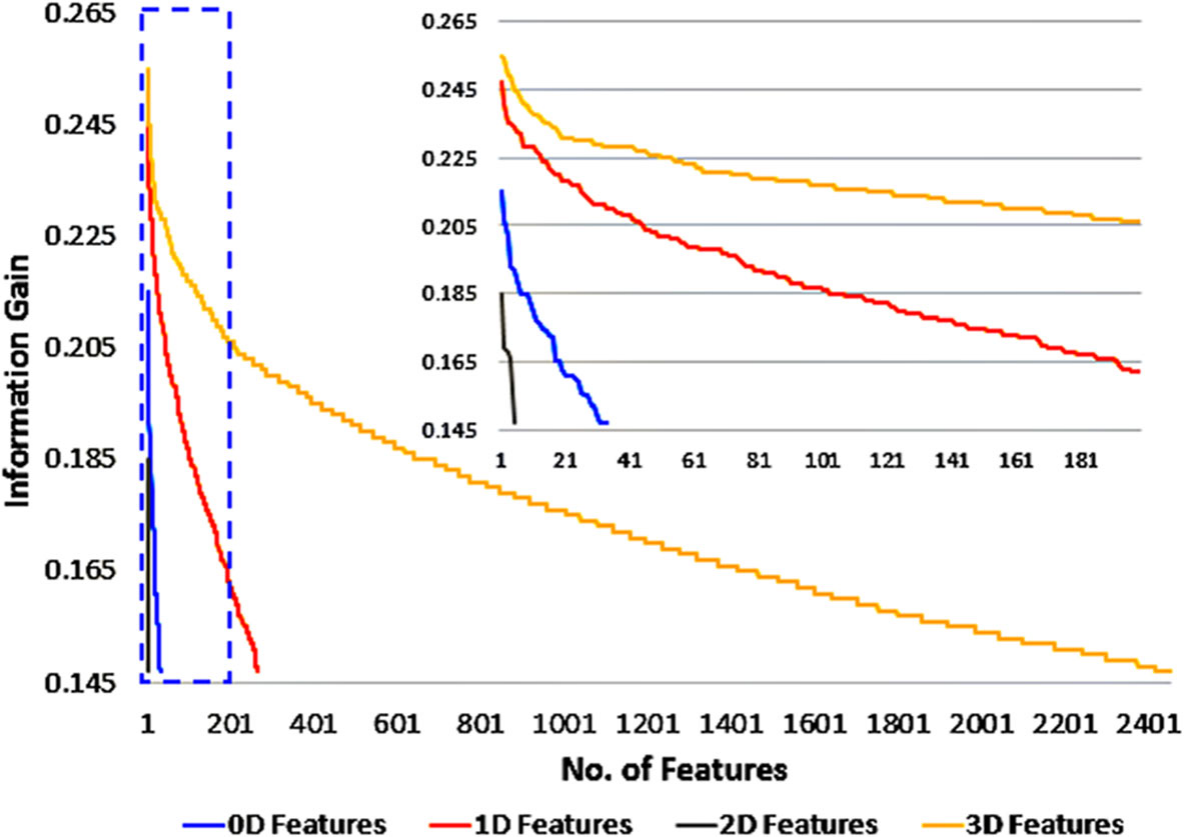
Information gain of the features of each protein family after redundancy reduction. Each point in the curves represents the number of descriptors (*x-axis*), of a given type, with IG value higher than its value (*y-axis*)

Figure 4 shows the graphical representations of the number of descriptors per value of IG for each descriptor family (0-3D) after selection by IG and redundancy reduction. This analysis illustrates the increase in the quality of the features from 0D to 3D types. This trend suggests that 3D–structural information is critical to obtain the most accurate discrimination between enzymes and non-enzymes. A recent article by Roche and Bruls [58] concluded that superfamily information is insufficient to determine the enzymatic nature of an unannotated protein, which supports the need to obtain a 3D–derived description of a protein for this task.

The gray curve (2D features) in Fig. 4 depicts the limited ability of this type of features to describe the present classification problem. This fact can be explained by the low relationship between the pseudo-fold 2D representations used here and the actual structural characteristics that determine the enzymatic nature of a protein. Given the low performance of the 2D features, for subsequent modelling steps only the 0D, 1D and 3D families are considered to build the final classifiers. Support Vector Machines (SVM) classification models are built using the different dimensional representations (0D, 1D and 3D) of the protein structure, based on the best subsets of features for each family.

Additional file 2: Table SI-12 summarizes the qualitative information associated with each of the extracted features from the three relevant descriptors families. This information provides some insights of the structural factors that determine the distinction between enzymes and non-enzymes proteins.

Three major structural characteristics are represented in the three sets: *i*) the presence of hydrophobic residues (a detailed analysis of the features, along the three descriptor classes, reveals the inclusion of specific aliphatic residues, such as isoleucine and leucine, as well as phenylalanine among non-polar aromatic residues); *ii*) the existence of polar residues; and *iii*) the presence of residues that promote reverse turns or secondary structure rupture. Such overarching structural features can be associated with the common globular type of the enzymes. The formation of a globular protein requires, on one hand, non-polar residues that form a stable hydrophobic core, and on the other hand, hydrophilic (polar) residues that stabilize the surface of the protein in a polar (aqueous) environment. In addition, in order to create such globular structure, tight turns and secondary-structure-ending points are also needed to permit the folding into a compact non-extended conformation. Glycine-associated features are extracted in addition to those related to residues promoting tight turns. This finding is supported by the results of [59], which, in an analysis of the hydrogen bonds present in catalytic sites, concluded that glycine constitutes 44% of the studied catalytic residues showing backbone–backbone interactions. This can be explained considering its small size making it easy to fit into a cavity within the active site architecture. The backbone amino (N–H) and carboxyl (C = O) groups of glycine are more accessible than those of bulkier amino acid residues, which are often occluded by the side-chain or their positions within secondary structure elements. Additionally, it has been previously suggested that glycine residues permit the enzyme active sites to change their structural conformations [60].

The presence of arginine- and histidine-associated descriptors also prevails as a strong structural feature associated with the enzymatic nature of a protein. Bartlett et al. found that the side-chains of these residues participate in more hydrogen bonds with a ligand than any other type of amino acids [59]. These authors examined the frequency of participation for each type of residue in nine different catalytic mechanisms: acid-base, nucleophile, transition state stabilizer, activate water, activate cofactor, primer, activate substrate, formation of radicals and chemically modified [59]. Then they construct a frequency chart with the occurrence of each type of residue in each of these classifications during catalysis [59]. The results show that histidine, in addition of being the most common residue in the studied active sites, is ubiquitous among all types of mechanisms. Besides, it is the residue with highest frequency of participation in general acid–base catalysis (51.3% of the appearances) which is recognized as the most frequent catalysis mechanism together with the transition state stabilizers [61]. Considering these two mechanisms together, histidine has a combined frequency of 67.3%, which is the second highest combined frequency among the most common types of residues found in the actives sites. Remarkably, in agreement with the extracted features in our models, arginine was identified as the residue with the highest combined frequency of participation in the two largest mechanisms, with a frequency of 83.8%. However, conversely to histidine this residue is most commonly involved the stabilization of the transition states (frequency of 75%). Taken together, histidine and arginine represent a 29.4% of the catalytic residues analyzed by [59], which is higher than the occurrence of any other pair of different residues including the negative ones, aspartate and glutamate, which have a population of 25.8%. In summary, these analyses support histidine- and arginine-associated descriptors as being strong determinants of the discrimination between enzymes and non-enzymes proteins.

### Identifying enzymes within the twilight zone using SVMs

SVM is a robust and widely used machine learning technique, with demonstrated effectiveness across dissimilar problems. For this particular classification challenge, the D&D dataset has been used previously as a gold-standard set to validate novel graph kernel approaches for SVM [33, 62–71]. Thus, we can compare our SVM-based models versus those previously reported for this data.

We use the Pearson VII Universal Kernel (PUK) function for building the SVM classifiers, because of the proven higher mapping power of this kernel related to more standard choices like Polykernel or radial basis function (RBF). Baydens et al. discussed precisely the suitability of this kernel when one does not have a priori knowledge of the nature of the data. These authors claim that the PUK function provides a more generalized approach than other kernels [72]. The PUK function has also been applied successfully to model other protein-related problems [73–76].

The tuning process for selecting the specific parameters of the SVM and kernel (C, omega and sigma) is described in Additional file 3.

#### Results using sequence-based (0-2D) features

The seminal article of D&D [33] presented the performance of a 0D model trained with the 20 amino acid composition frequencies as the descriptors for the protein structures in the dataset. The authors reported an accuracy in 10-fold cross-validation of 74.83 ± 1.37% using a SVM with a RBF kernel. Here, the nine 0D descriptors resulting from the features selection process were used to train a SVM model using a penalty parameter (C = 8) and the PUK with omega and sigma parameters equal to 21 and 7 respectively.

In a similar way, the extracted set of 1D descriptors was used to train a SVM model (C = 0.5, omega = 1, sigma = 1). The outcome probability estimate was tuned using logistic regression models. The resulting accuracy in 10-fold cross validation was 78.83 ± 0.21%, which is significantly higher than that obtained using 0D features. Remarkably, such performance surpasses several of the 3D methods previously evaluated on the D&D dataset (see Table 2). This result validates the relevant capability of 1D sequence-based descriptors generated with ProtDCal to properly describe fundamental characteristics that determine the enzymatic nature of a given protein.

**Table 2:** Comparison with published results, in 10-fold cross-validation, of SVM methods using the D&D dataset

Kernel	Accuracy* (%)	Reference	Run time	Computer
PUK	82.0 ± 0.3	ProtDCal 3D model	53 m 2 s	Intel Core i5–3210 M 2.5 GHz with 8 GB of RAM
GraphK ShinglingWL	81.54 ± 1.54	[62]	3 h 1 m 7 s	Apple MacPro with 3.0GHz Intel 8-Core with 16GB RAM
GraphK WLmod	80.31	[63]	25 m 0 s	NA
Radial	80.17 ± 1.24	[33]	NA	NA
GraphK WL	79.78 ± 0.36	[64]	11 m 0 s	Apple MacPro with 3.0GHz Intel 8-Core with 16GB RAM
GraphK WL	79.00 ± 0.2	[65]	6 m 42 s	3.4GHz Intel core i7 processors
PUK	78.8 ± 0.2	ProtDCal 1D model	3 m 42 s	Intel Core i5–3210 M 2.5 GHz with 8 GB of RAM
GraphK WL	78.29	[66]	2 h 12 m 57 s	MAC OS × 10.5 with two 2.66GHz Dual Core Intel Xeon processors, with 4GB 667MHz DDR2 memory
PUK	77.58	[68]	21 m 51 s	2.5 GHz Intel 2-Core processor (i.e. i5–3210 m)
GraphK LWL	76.60 ± 0.6	[69]	11 m 00s	16 cores machine (Intel Xeon CPU E5–2665@2.40GHZ and 96GB of RAM)
GraphK SP	75,87	[70]	1 h 40 m 57 s	NA
GraphK PRW	75.40 ± 0.6	[71]	NA	NA

The runtimes reported for our models comprise both the time for computing the features and times related to the building and assessing the models using Weka 3.7.11

*NA* Not-available

*For each of the listed references, the tabulated accuracy corresponds to the best performance in the D&D dataset as shown in the article

Runtime and computational resource were also displayed for the methods included in the comparison

All the referenced methods constitute 3D classifiers given that they use 3D–graphs to represent the protein structure

The final five features extracted from the 2D family of descriptors were also used to train a SVM classifier (C = 64, omega = 1, sigma = 1). Unfortunately, as the IG analysis showed, the information content encoded by these features is not highly related with the intrinsic characteristic that differentiates enzyme from non-enzyme proteins. The obtained accuracy in 10-fold cross-validation was only of 71.86%, which is lower than the performance of 0D features shown above. Such results indicate that the Nandy’s and FCM pseudo-fold representations are not suitable for the modelled problem and may introduce noisy information that limits the capability to train an accurate classifier.

#### Results using 3D–structure features

The set of 26 3D descriptors previously extracted, was used to train a SVM model (C = 2, omega = 11 and sigma = 2). Again, here logistic regression was used to estimate of the outcome probabilities. A 10-fold cross-validation test resulted in an accuracy of 82.00 ± 0.32%. Table 2 summarizes the.

performance, runtime and computational resource for several 3D methods that were trained and assessed using the D&D dataset. Table 2 also included these measures for the most significant ProtDCal-based models (1D and 3D–based) as well as the best predictive SVM model shown by Dobson and Doig using their 3D–structure features [34].

Most of these methods use graphical kernels in order to manage the 3D–graph representations for protein structures. The graphs are formed by assuming the presence of an edge when a pair of residues is found below a given cut-off of spatial separation. An earlier study of Li et al. proposed that, for classification problems based on large graphs, instead of relying on patterns such as path, cycles, sub-trees, and sub-graphs, a valid approach would be to instead construct a feature vector for graph classification [66]. They used 20 topological features derived from each graph (protein) to train a SVM model with a Gaussian kernel. They obtained a rather similar accuracy (76.32 ± 2.72%) than that showed by methods using graph kernels [66], however, their approach supports the use of 3D structure-based features for modeling the enzyme vs. non-enzyme discrimination problem.

Remarkably, our method outperforms all the models described in the literature using the D&D dataset to train and assess their predictors. Furthermore, we evaluated the prediction accuracy of our best model (using 3D–structure-based protein descriptors) in the same hold-out dataset, used by D&D in their original work. This separate subset is composed of 52 proteins, structurally unrelated to the training dataset. We achieved in this test set an accuracy of 80.8% while D&D obtained 79.0%. Our higher accuracy, together with its similarity to that obtained in CV, prove the superiority of our model as well as the absence of a possible overfitting during the training and CV of the model.

We remark that the results presented in this report were produced by using general-purpose features, i.e. no problem-specific (ad-hoc) modifications were carried out to the features. Such performance validates the applicability of the feature generation strategy implemented in ProtDCal, which differs from other methods in that it splits the structural information into dissimilar packages (descriptors) either with global or local information. Such *divide-and-conquer* approach permits one to extract the most relevant features, following a supervised feature selection process, and to neglect noisy or irrelevant information present in the protein structure.

On the other hand, the analysis of the run times summarized in the Table 2 evidences that our 3D model displays similar computational cost to the other methods applied to the same dataset. Nonetheless, the sequence-based (1D) model shows a significantly lower runtime than the other methods. This fact is particularly relevant because the sequence-based model has a wider domain of application and at the same times it reaches a similar performance to other 3D–based methods.

Hence, altogether, the results presented above confirm the use of ProtDCal for generating information-rich features capable of describing key structural characteristics of proteins, which determine their specific functions. At the same time, we introduce 2 AF methods, one based on primary structure features (1D) and the other based on 3D structures, which can be valuable tools for the prediction or classification of the enzymatic nature of proteins.

### Identifying enzymes among former uncharacterized proteins in the *Shewanella oneidensis* proteome

As the applicability of 3D–based models is limited by the availability of detailed structural information in protein data and by the computational cost implied in the estimation of 3D features, our alignment-free (AF) model based on 1D information (sequence) has a wider practical use to identify enzymes from proteome databases. Proteins of unknown function comprise 30–40% of the proteins in annotated proteomes. Therefore, assigning a biological role to these proteins is a challenge that often cannot be reliably addressed by alignment algorithms. Under this scenario, AF approaches are more suitable to provide clues about the function of uncharacterized proteins in proteomes. Thus, homology-independent models/methodologies that distinguish enzymes and non-enzymes can effectively guide experimentalists toward accurate annotation of protein function. Here, we present a case study represented by a subset of 30 proteins identified as “uncharacterized proteins” during the proteome annotation of the bacterium *Shewanella oneidensis* in 2002 [77]. These proteins were selected since they were later extensively annotated by Louie, B et al. in 2008, creating a benchmark annotation dataset [34]. The annotation of this dataset resulted in 23 validated enzymes and 7 non-enzyme proteins. We use this benchmark dataset to comparatively evaluate the classification performance of methods identifying enzyme-like proteins (ProtDCal-based-1D model, EzyPred [24] and EnzymeDetector [12]) on former uncharacterized proteins that now are accurately annotated. Table 3 shows the success rates in identifying the enzyme and non-enzyme proteins on the benchmark annotation dataset (30 formerly uncharacterized proteins from the *S. oneidensis* proteome). Detailed information about the benchmark annotation dataset and the prediction performed for each method/protein is summarized in Additional file 2: Table SI-13.

**Table 3:** Success rates of sequence-based enzyme identification methods on the benchmark dataset made up of 30 formerly uncharacterized proteins from the *S. oneidensis* proteome

	Number of correct predictions	Success rate (%)
ProtDCal-1D-model	23	76.67
EzyPred	16	53.33
EnzymeDetector	27	90.00

Our sequence-based-1D model showed a higher accuracy than EzyPred. This is despite the fact that the EzyPred is a powerful classification engine, based on optimized evidence-theoretic K-nearest-neighbour (OET-KNN) classifiers, which are trained with the comprehensive ENZYME repository (http://www.expasy.org/enzyme/) and considers functional domains and evolutionary information for the enzymes identification [78].

On the other hand, the EnzymeDetector tool is one of the most popular methodologies [12] for assigning enzymatic function by sequence similarity search in BRENDA, which in turn is the main information system of functional, biochemical and molecular enzyme data [79]. Given the proteome of *Shewanella oneidensis* is already annotated in BRENDA, it is expected that a similarity-based approach like EnzymeDetector must show essentially a perfect classification performance (100%) among these proteins. However, this method still did not recognize three benchmarked enzymes with the following locus IDs: SO_2603, SO_3578 and SO_4680 (Additional file 2: Table SI-13) that, remarkably, our method was able to predict properly. Surprisingly, these three cases are not integrated in BRENDA, which is an evidence that whenever is possible, functional predictions should not be based only on sequence similarities; they should be confirmed from methods of different background.

This retrospective prediction study on uncharacterized proteins confirms the applicability of our models, and therefore of ProtDCal’s general-purpose descriptors for developing machine learning models for protein functions prediction.

## Conclusions

In summary, we present a model based on 3D–structure features that ranks on the top of the SVMs-based methods of enzyme identification according the performance in the gold-standard D&D dataset. An alignment-free model using primary-structure-based descriptors (1D) was developed, achieving first comparable results with other 3D–structure-based methods and also higher performance than the sequence-based method EzyPred in distinguishing enzymes from non-enzymes within a set of proteins of *S. oneidensis*.

Our protein descriptors, implemented in ProtDCal, are meant to be a powerful protein encoding platform for data mining of structurally dissimilar protein-related data. The fundamental basis of the general-purpose nature of ProtDCal is its *divide-and-conquer* codification scheme, which followed by supervised features selection, can eliminate irrelevant or noisy structural information and focus the input learning data in the key features that can be correlated with a determine function or property.

## Additional files

Additional file 1: Different families of AF protein predictors implemented in ProtDCal and TI2BioP. (PDF 136 kb)

Additional file 2: Supplementary figures (**Figure SI-I** and **SI-2**) and Tables (**Table SI-1** to **Table SI-13**). **Figure SI-1** Dot plot of the pairwise amino acid identity matrix expressed in percentage (colour bar) for the D&D dataset. (A) Global all-vs-all sequence alignments using the Needleman-Wunsch (NW) algorithm (B) Local all-vs-all sequence alignments using the Smith-Waterman (SW) algorithm. **Figure SI-2** Structural diversity summary of the D&D dataset according to SCOP database. **Table SI-1** Compendium of structural and chemical-physical amino acid properties. **Table SI-2** Formulae and description of Thermodynamics Indices for Protein Sequences. **Table SI-3** Formulae and description of Topographic Indices. **Table SI-4** Formulae and description of 3D–Thermodynamics Indices. **Table SI-5** Summary of the definitions of amino acid groups. **Table SI-6** Aggregation operators: Norms (Metrics) Invariants. **Table SI-7** Aggregation operators: Mean (First Statistical Moment) Invariants. **Table SI-8** Aggregation operators: Statistical (Highest Statistical Moments) Invariants. **Table SI-9** Aggregation operators: Information-Theory-based Invariants. **Table SI-10** Structural diversity summary of the D&D enzyme subset according to SCOP hierarchal database. **Table SI-11** Structural diversity summary of the D&D enzyme subset according to SCOP hierarchal database. **Table SI-12** Structural information of the selected ProtDCal’s features from the different families of descriptors (0D, 1D & 3D). **Table SI-13** Detailed information about the benchmark annotation dataset and the prediction performed for each method/protein. Misclassified cases are highlighted in red font. (PDF 1850 kb)

Additional file 3: Experiments leading to the selection of the SVM and kernel parameters. (PDF 310 kb)

## Abbreviations

AB: Alignment-based
AF: Alignment-free
D&D: Dobson and Doig
IG: Information gain
ProtDCal: Protein Descriptor Calculation
PseAAC: Pseudo amino acid composition
PUK: Pearson VII function-based universal kernel
SVMs: Support vector machines
TI2BioP: Topological Indices to BioPolymers
