# Supplementary material for: Exploring general-purpose protein features for distinguishing enzymes and non-enzymes within the twilight zone
Source: BMC Bioinformatics. 2017 Jul 21;18:349. doi: 10.1186/s12859-017-1758-x (PMC5521120; doi:10.1186/s12859-017-1758-x)
Supplement: Supplementary file 1 — Different families of AF protein predictors implemented in ProtDCal and TI2BioP. (PDF 136 kb) [file 12859_2017_1758_MOESM1_ESM.pdf]

Additional File-1

Different families of AF protein predictors implemented in ProtDCal and TI2BioP

Compositional features (0D). The classic example of this family of protein features are the amino acid composition frequencies of a protein. ProtDCal generalizes these frequencies into a variety of features through the application of invariant-aggregation operators (iAO). The iAO can be applied to the entire protein or to groups of residues selected according to their properties, i.e. residues which are polar, hydrophobic, aromatic, etc. The iAO constitute norms, central-tendency, dispersion and information-theoretic measures, which do not depend on the ordering of residues within the group.

Linear-topology-based features (1D). This family comprises protein descriptors considering information from the ordering of residues in the protein. The most common examples of this type of features are the Pseudo-Amino acid Composition indices introduced by Chou *et al* and implemented in their server PseAAC [1]. ProtDCal allows the generation of this type of feature by extending topological operators, traditionally applied to compute molecular descriptors for small and mid-sized organic molecules [2], to the analysis of proteins. Examples of these operators include the Electrotopological State [3], the Kier-Hall connectivity indices [4] and the Moreau-Broto autocorrelation [5]. Such operators are used to modify the intrinsic value of an index for a given residue according to its neighbours at a given topological distance (sequence separation).

Pseudo-fold-based features (2D). 2D protein AF descriptors were estimated by applying the graph-theory to 2D artificial representations of DNA and protein sequences, i.e. Nandy or Cartesian representation [6, 7], and Four-Colour maps [8, 9], in analogy to topological descriptors (spectral moments) defined by Estrada for small-sized organic molecules [10]. TI2BioP considers the “building blocks” of DNA/RNA and protein

biopolymers as nodes and the bonds between them as edges in 2D graphs. The information contained in a biopolymer chain is simplified in the topology of a 2D graph that is determined by the sequence order and the nucleotide/amino acid composition of the biopolymer. Thus, the information encoded by the spectral moments will largely depend on the representation used to compute the descriptors. These pseudo-folding 2D descriptors have been used successfully to detect distant similarities among sequences with low homology level [9, 11]. However, the general applicability of these descriptors are affected by the arbitrary nature of the assumed topology; while a given artificial structure may be relevant to model a specific problem, this same structure may not be useful in another problem. The most significant relevancy of this family is that, whenever one is capable of defining a meaningful topology for amino acid sequences, it is then possible to compute features directly from the sequences but including further information content not encoded by a simple linear topology.

Structure-based features (3D). In general, 3D-structure features arise from various theoretical underpinnings, including polymer-based descriptors such as the gyration radius, the end-to-end distance, solvent-accessible surface area, and the Estrada's folding degree [12, 13]. In addition, there are a number of contact-based descriptors that were introduced in order to describe the relationship between the folding-rate constant and the native protein structure, e.g. the Contact Order [14], Long Range Order [15], and Total Contact Distance [16], etc. A third family of descriptors comes from applying concepts of Graph Theory [17] for defining descriptors of the protein topology. Such features use inter-residue contacts to define new edges in the topological graph, thus obtaining a description of the 3D structure. Some examples of graph-based descriptors are the average degree, average clustering coefficient, average path length, etc. [18]. In addition, graph-derived features have been introduced by González-Díaz *et al.*, which extended

algebraic topological (2D) descriptors, based on spectral moments, by applying 2D projections of the protein 3D structure [19-22]. All together, these 3D features lead to only a few hundred descriptors, which is contradictory with the complexity and size of protein 3D structures when one considers the hundreds of thousands of descriptors defined for small and mid-sized organic molecules, as summarized in the Handbook of Molecular Descriptors [2], which ranges in the order of hundreds of thousands. ProtDCal was introduced as an attempt to address this shortfall through its capability to generate thousands of 3D structural descriptors for a given protein. ProtDCal implements the above-mentioned contact-based descriptors with proven relationship to the folding kinetic and stability parameters (see Table S3 in Additional file 2). In addition, the program introduces thermodynamic features derived from a coarse-grained model developed to estimate the most relevant free-energy contributions to protein folding stability [23-25] and the folding rate constant [26].

References

1. Shen HB, Chou KC: **PseAAC: a flexible web server for generating various kinds of protein pseudo amino acid composition.** *Anal Biochem* 2008, **373**:386–388.
2. Todeschini R, Consonni V: **Frontmatter**. In: *Handbook of Molecular Descriptors.* Wiley-VCH Verlag GmbH; 2008: i-xxi.
3. Kier LB, Hall LH: **An Electrotopological-State Index for Atoms in Molecules**. *Pharm Res* 1990, **7**:801-807.
4. Kier LB, Hall LH: **Molecular Connectivity in Structure-Activity Analysis**. Chichester (UK): Research Studies Press - Wiley; 1986.
5. Moreau G, Broto P: **The Autocorrelation of a topological structure. A new molecular descriptor.** *Nouv J Chim* 1980, **4**:359-360.
6. Nandy A: **Two-dimensional graphical representation of DNA sequences and intron-exon discrimination in intron-rich sequences**. *Comput Appl Biosci* 1996, **12**(1):55-62.
7. Aguero-Chapin G, Gonzalez-Diaz H, Molina R, Varona-Santos J, Uriarte E, Gonzalez-Diaz Y: **Novel 2D maps and coupling numbers for protein sequences. The first QSAR study of polygalacturonases; isolation and prediction of a novel sequence from Psidium guajava L**. *FEBS Lett* 2006, **580**(3):723-730.
8. Randić M, Mehulić K, Vukičević D, Pisanski T, Vikić-Topić D, Plavšić D: **Graphical representation of proteins as four-color maps and their numerical characterization**. *J Mol Graphics Modell* 2009, **27**(5):637-641.
9. Agüero-Chapin G, Molina-Ruiz R, Maldonado E, de la Riva G, Sánchez-Rodríguez A, Vasconcelos V, Antunes A: **Exploring the adenylation domain**

repertoire of nonribosomal peptide synthetases using an ensemble of sequence-search methods. *PLoS ONE* 2013, **8**(7).
10. Estrada E: **Spectral Moments of the Edge Adjacency Matrix in Molecular Graphs. 1. Definition and Applications to the Prediction of Physical Properties of Alkanes**. *J Chem Inf Comput Sci* 1996, **36**:844-849.
11. Aguero-Chapin G, Perez-Machado G, Molina-Ruiz R, Perez-Castillo Y, Morales-Helguera A, Vasconcelos V, Antunes A: **TI2BioP: Topological Indices to BioPolymers. Its practical use to unravel cryptic bacteriocin-like domains**. *Amino Acids* 2011, **40**(2):431-442.
12. Estrada E: **Characterization of the folding degree of proteins**. *Bioinformatics* 2002, **18**(5):697-704.
13. Estrada E: **A Protein Folding Degree Measure and Its Dependence on Crystal Packing, Protein Size, Secondary Structure, and Domain Structural Class**. *J Chem Inf Comput Sci* 2004, **44**:1238-1250.
14. Plaxco KW, Simons KT, Baker D: **Contact Order, Transition State Placement and the Refolding Rates of Single Domain Proteins**. *J Mol Biol* 1998, **277**:985-994.
15. Gromiha MM, Selvaraj S: **Comparison between long-range interactions and contact order in determining the folding rate of two-state proteins: application of long-range order to folding rate prediction**. *J Mol Biol* 2001, **310**:27-32.
16. Zhou H, Zhou Y: **Folding Rate Prediction Using Total Contact Distance**. *Biophysical Journal* 2002, **82**:458–463.
17. Bittner L: **C. Berge, Théorie des graphes et ses applications. VIII + 277 S. m. 117 Abb. Paris 1958. Dunod Editeur. Preis geb. 3400 F**. *ZAMM - Journal of Applied Mathematics and Mechanics / Zeitschrift für Angewandte Mathematik und Mechanik* 1960, **40**(5-6):281-281.
18. Li G, Semerci M, Yener B, Zaki MJ: **Effective graph classification based on topological and label attributes**. *Stat Anal Data Min* 2012, **5**(4):265-283.
19. González-Díaz H, Molina R, Uriarte E: **Stochastic molecular descriptors for polymers. 1. Modelling the properties of icosahedral viruses with 3D-Markovian negentropies**. *Polymer* 2004, **45**:3845–3853.
20. González-Díaz H, Saíz-Urra L, Molina R, Uriarte E: **Stochastic molecular descriptors for polymers. 2. Spherical truncation of electrostatic interactions on entropy based polymers 3D-QSAR** *Polymer* 2005, **46**:2791–2798.
21. González-Díaz H, Molina R, Uriarte E: **Recognition of stable protein mutants with 3D stochastic average electrostatic potentials**. *FEBS LETTERS* 2005, **579**:4297–4301.
22. González-Díaz H, Saiz-Urra L, Molina R, Santana L, Uriarte E: **A model for the recognition of protein kinases based on the entropy of 3D van der Waals interactions**. *J Proteome Res* 2007, **6**:904–908.
23. Ruiz-Blanco YB, Marrero-Ponce Y, Paz W, García Y, Salgado J: **Global Stability of Protein Folding from an Empirical Free Energy Function.** *Journal of Theoretical Biology* 2013, **321**:44-53.
24. Ruiz-Blanco YB, Marrero-Ponce Y, García Y, Puris A, Bello R, Green J, Sotomayor-Torres CM: **A physics-based scoring function for protein structural decoys:Dynamic testing on targets of CASP-ROLL**. *Chemical Physics Letters* 2014, **610–611**:135–140.
25. Ruiz-Blanco YB, García Y, Sotomayor-Torres CM, Marrero-Ponce Y: **New Set of 2D/3D Thermodynamic Indices for Proteins. A Formalism Based on "Molten Globule" Theory**. *Physics Procedia* 2010, **8**:63-72.
26. Ruiz-Blanco YB, Marrero-Ponce Y, Prieto PJ, Salgado J, García Y, Sotomayor-Torres CM: **A Hooke′ s law-based approach to protein folding rate**. *Journal of theoretical biology* 2015, **364**:407-417.
