# Supplementary material for: Exploring general-purpose protein features for distinguishing enzymes and non-enzymes within the twilight zone
Source: BMC Bioinformatics. 2017 Jul 21;18:349. doi: 10.1186/s12859-017-1758-x (PMC5521120; doi:10.1186/s12859-017-1758-x)
Supplement: Supplementary file 2 — Supplementary figures (Figure SI-I and SI-2) and Tables (Table SI-1 to Table SI-13). Figure SI-1 Dot plot of the pairwise amino acid identity matrix expressed in percentage (colour bar) for the D&D dataset. (A) Global all-vs-all sequence alignments using the Needleman-Wunsch (NW) algorithm (B) Local all-vs-all sequence alignments using the Smith-Waterman (SW) algorithm. Figure SI-2 Structural diversity summary of the D&D dataset according to SCOP database. Table SI-1 Compendium of structural and chemical-physical amino acid properties. Table SI-2 Formulae and description of Thermodynamics Indices for Protein Sequences. Table SI-3 Formulae and description of Topographic Indices. Table SI-4 Formulae and description of 3D–Thermodynamics Indices. Table SI-5 Summary of the definitions of amino acid groups. Table SI-6 Aggregation operators: Norms (Metrics) Invariants. Table SI-7 Aggregation operators: Mean (First Statistical Moment) Invariants. Table SI-8 Aggregation operators: Statistical (Highest Statistical Moments) Invariants. Table SI-9 Aggregation operators: Information-Theory-based Invariants. Table SI-10 Structural diversity summary of the D&D enzyme subset according to SCOP hierarchal database. Table SI-11 Structural diversity summary of the D&D enzyme subset according to SCOP hierarchal database. Table SI-12 Structural information of the selected ProtDCal’s features from the different families of descriptors (0D, 1D & 3D). Table SI-13 Detailed information about the benchmark annotation dataset and the prediction performed for each method/protein. Misclassified cases are highlighted in red font. (PDF 1850 kb) [file 12859_2017_1758_MOESM2_ESM.pdf]

Additional File-2

Figures

- **Figure SI-1.** Dot plot of the pairwise amino acid identity matrix expressed in percentage (colour bar) for the D&D dataset. (A) Global *all-vs-all* sequence alignments using the Needleman-Wunsch (NW) algorithm (B) Local *all-vs-all* sequence alignments using the Smith-Waterman (SW) algorithm.
- **Figure SI-2.** Structural diversity summary of the D&D dataset according to SCOP database.

Tables

- **Table SI-1**. Compendium of structural and chemical-physical amino acid properties.
- **Table SI-2**. Formulae and description of Thermodynamics Indices for Protein Sequences.
- **Table SI-3**. Formulae and description of Topographic Indices.
- **Table SI-4**. Formulae and description of 3D-Thermodynamics Indices.
- **Table SI-5**. Summary of the definitions of amino acid groups.
- **Table SI-6**. Aggregation operators: Norms (Metrics) Invariants.
- **Table SI-7**. Aggregation operators: Mean (First Statistical Moment) Invariants.
- **Table SI-8**. Aggregation operators: Statistical (Highest Statistical Moments) Invariants.
- **Table SI-9**. Aggregation operators: Information-Theory-based Invariants.
- **Table SI-10.** Structural diversity summary of the D&D enzyme subset according to SCOP hierarchal database.
- **Table SI-11.** Structural diversity summary of the D&D enzyme subset according to SCOP hierarchal database.
- **Table SI-12.** Structural information of the selected ProtDCal's features from the different families of descriptors (0D, 1D & 3D)
- **Table SI-13**. Detailed information about the benchmark annotation dataset and the prediction performed for each method/protein. Misclassified cases are highlighted in red font

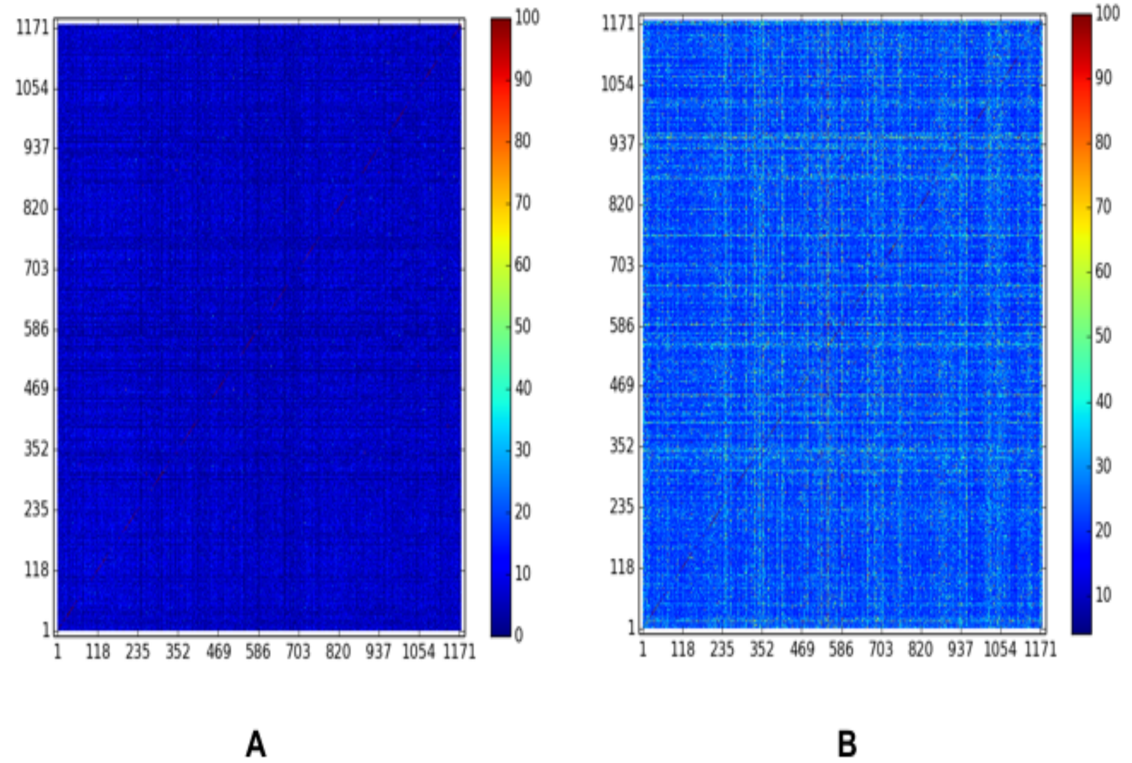

Figure SI-1. Dot plot of the pairwise identity matrix expressed in percentage (colour bar) for the D&D dataset. (A) Global *all-vs-all* sequence alignments using the Needleman-Wunsch (NW) algorithm (B) Local *all-vs-all* sequence alignments using the Smith-Waterman (SW) algorithm.

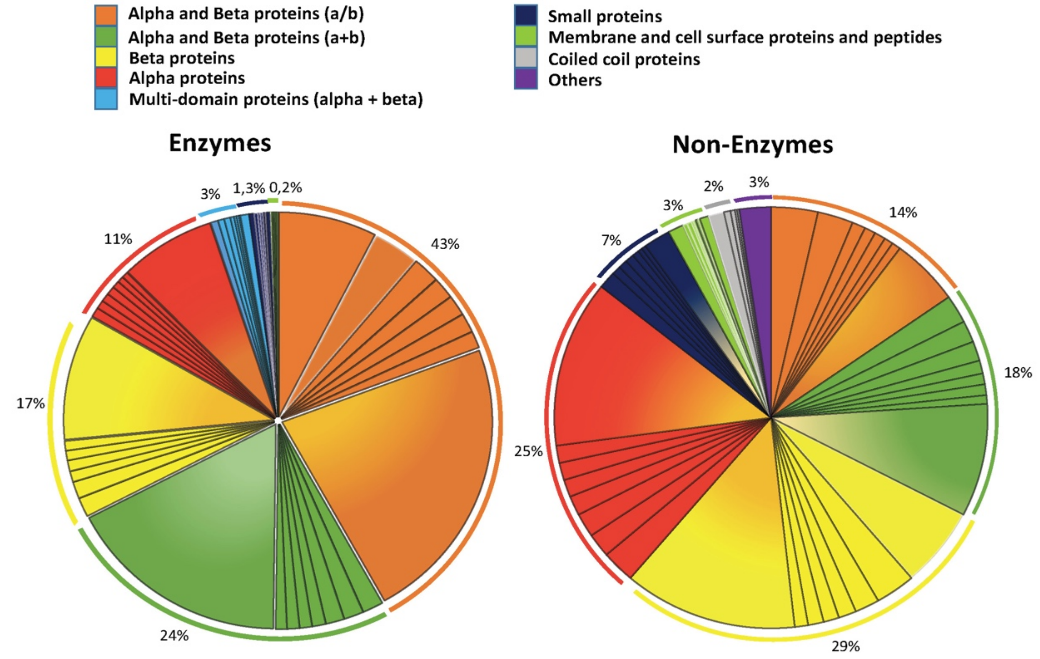

Figure SI-2. Structural diversity summary of the D&D dataset according to SCOP database. Main structural classes are labelled using the assigned colours in the legend. Each class is subdivided by lines into subsections corresponding to the structural folds. Graph subsections (Folds) represented by colour gradients are made up of several fold types. These subsections are termed as "others". The information provided in the figure is complemented with the data shown in supplementary tables SI-1 and SI-2.

Table SI-1. Compendium of structural and chemical-physical amino acid properties.

	Mw	HP	IP	ECI	ISA	Z1	Z2	Z3	Pb	Pt	Pa
**ALA**	71	1.8	6.01	0.05	62.9	0.07	-1.73	0.09	0.9	0.78	1.29
**ARG**	156	-4.5	10.76	1.69	52.98	2.88	2.52	-3.44	0.99	0.88	0.96
**ASN**	114	-3.5	5.41	1.31	17.87	3.22	1.45	0.84	0.76	1.28	0.9
**ASP**	115	-3.5	2.77	1.25	18.46	3.64	1.13	2.36	0.72	1.41	1.04
**CYS**	103	2.5	5.07	0.15	78.51	0.71	-0.97	4.13	0.74	0.8	1.11
**GLN**	128	-3.5	3.22	1.31	19.53	3.08	0.39	-0.07	0.75	1	1.44
**GLU**	129	-3.5	5.65	1.36	30.19	2.18	0.53	-1.14	0.8	0.97	1.27
**GLY**	57	-0.4	5.97	0.02	19.93	2.23	-5.36	0.3	0.92	1.64	0.56
**HIS**	137	-3.2	7.59	0.56	87.38	2.41	1.74	1.11	1.08	0.69	1.22
**ILE**	113	4.5	6.02	0.09	149.77	-4.44	-1.68	-1.03	1.45	0.51	0.97
**LEU**	113	3.8	5.98	0.01	154.35	-4.19	-1.03	-0.98	1.02	0.59	1.3
**LYS**	128	-3.9	9.74	0.53	102.78	2.84	1.41	-3.14	0.77	0.96	1.23
**MET**	131	1.9	5.74	0.34	132.22	-2.49	-0.27	-0.41	0.97	0.39	1.47
**PHE**	147	2.8	5.48	0.14	189.42	-4.92	1.3	0.45	1.32	0.58	1.07
**PRO**	97	-1.6	6.48	0.16	122.35	-1.22	0.88	2.23	0.64	1.91	0.52
**SER**	87	-0.8	5.68	0.56	19.75	1.96	-1.63	0.57	0.95	1.33	0.82
**THR**	101	-0.7	5.87	0.65	59.44	0.92	-2.09	-1.4	1.21	1.03	0.82
**TRP**	186	-0.9	5.89	1.08	179.16	-4.75	3.65	0.85	1.14	0.75	0.99
**TYR**	163	-1.3	5.66	0.72	132.16	-1.39	2.32	0.01	1.25	1.05	0.72
**VAL**	99	4.2	5.97	0.07	120.91	-2.69	-2.53	-1.29	1.49	0.47	0.91

**Mw**	Molar Weight (12)
**HP**	Kyte-Doolitle's Hydrophobicity Scale (5)
**IP**	Isoelectric Point (12)
**ECI**	Electronic Charge Index (6)
**Z1**	Composed parameter related with hydrophilicity (14)
**Z2**	Composed parameter related with steric features (14)
**Z3**	Composed parameter related with electronic features (14)
**ISA**	Isotropic Surface Area (6)
**Pa**	Levitt's Probability of adopting alpha helix conformation (15)
**Pb**	Levitt's Probability of adopting beta sheet conformation (15)
**Pt**	Levitt's Probability of adopting beta turn conformation (15)

Table SI-2. Formulae and description of Thermodynamics Indices for Protein Sequences.

Acronym	Formula	Description
W(U)	$W_i^U = \;\; {}_{j=i\;\;2}^{i+2} N_j^w$	Number of water molecules close to a residue in an unfolded state (3).
Gw(U)	$G_w(U)_i = \;\; TR \;\; {}_{hyd} \ln \frac{W_i^U!}{(W_i^U \;\; N_i^w)!}$	Free energy contribution from the entropy of the first shell of water molecules in an unfolded state (3).
Gs(U)	$G_s(U)_i = H_i A_i^U$	Interfacial free energy contribution of an unfolded state

Table SI-3. Formulae and description of 3D-structure indices.

Acronym	Formula	Description
A_F	-	Solvent accessible surface area
ΔA	$\Delta A = A_F - A_U$	Buried area. Where A_U is the fully exposed surface area of each residue and A_F is the area in the folded state.
ΔA^{np}	$\Delta A^{np} = A^{np}{}_F - A^{np}{}_U$	Buried non-polar area. Here nitrogen atoms and oxygen atoms are excluded.
wSp	$wSp_i = {}_i * {}^{s}_{i}$	Weighted index of the solvent accessibility.<br>Where ω represents any weighting property and the delta takes value 1 or 0 if the residue is considered superficial or internal respectively.
lnFD	$\ln FD_i = \frac{{}^{N}_{j;\|j\ \ i\|>1}\ \|j\ \ i\|/d_{ij}^3}{N\ \ x}$	Logarithm of the Folding Degree. Where *d* is the spatial distance, *N* the length of the protein and *x* a parameter which takes value 2 for terminal residues and 3 for the others.
wR^2	$wRG_i^2 = \frac{w_i * d_i^2}{{}^{N}_{i=1}\ w_i}$	Weighted Squared Radius. Where ω represents any weighting property and *d* is the spatial distance.
wΔHBd	$Hbd_i = {}_i *({}_N + {}_O)$<br>Geometric definition of a H-bond:<br>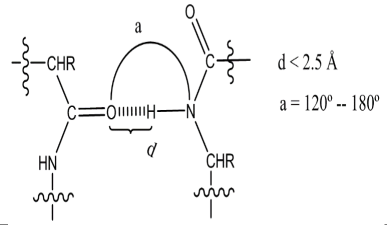 	Weighted deficit or excess of the H-bond between the backbone atoms. Where ${}^{N}_{ij}$ takes value 1 if the nitrogen atom of residue *i* is buried ($A_{(N)}$ < 0.01Å) and is not H-bonded with any oxygen atom or 0 otherwise. In the same way ${}^{O}_{ij}$takes value 1 if the oxygen atom of residue *i* is buried ($A_{(O)}$ < 0.01Å) and is not H-bonded with any nitrogen atom and 0 otherwise.
wNc	$wNc_i = 0.5\ {}^{N}_{j\ \ i}\ {}_{ij}\ {}^{c}_{ij}$	Weighted Number of Contact. Where δ_{ij}, takes value 1 when the contact conditions are fulfilled and 0 otherwise. A contacts is defined for pair of residues with spatial distances shorter than a cutoff *d* and topological distances longer than a cutoff *t*. The parameter ω_{ij} represents a weighting coefficient for each pair of residues. This parameter is computed as the product, $\omega_i\omega_j$, of the values, for each residue, of any property within a pool of 12 aminoacid properties covering structural, physical-chemical features.
wFLC	$wFLC_i = \frac{{}^{N}_{\|j\ \ i\|\ 4}\ {}_{ij}\ {}^{c}_{ij}}{{}^{N}_{i=1}\ {}^{N}_{j=1}\ {}_{ij}\ {}^{c}_{ij}}$	Weighted Fraction of Local Contancts. The parameters δ_{ij} and ω_{ij} means the same as previous but here the topological cutoff value is fixed in $t = 1$.
wNLC	$wNLC_i = 0.5\ {}^{N}_{\|j\ \ i\|\ 4}\ {}_{ij}\ {}^{c}_{ij}$	Weighted Number of Local Contact The parameters δ_{ij} and ω_{ij} means the same as in *wNc* but here the topological cutoff value is fixed in $t = 1$.
wCO	$wCO_i = \frac{1}{2NN_c}\ {}^{N}_{j\ \ i}\ {}_{ij}\ {}^{c}_{ij}$	Weighted Relative Contact Order (7). Where Nc represents the number of contacts in the protein.

wLCO	$wLCO_i = \frac{{}^{N}_{j\ i}\ {}_{ij}\ {}^{c}_{ij}}{N\ {}^{N}_{j\ i}\ {}^{c}_{ij}}$	Weighted Local Contact Order. As difference with previous, the weighted contacts are divided by the same un-weighted local contact instead of all the contact in the protein.
wRWCO	$wRWCO_i = \frac{{}^{N}_{j\ i}\ {}_{ij}\ {}^{c}_{ij}}{N}$	Weighted Residue-Wise Contact Order (8).
wCTP	$wCTP_i = \frac{1}{2NN_c}\ {}^{N}_{j\ i}\ {}^{2}_{ij}\ {}^{c}_{ij}$	Weighted Chain Topology Parameter (9).
wCLQ	$wCLQ_i = \frac{{}_{j<l}\ {}_{ij}\ {}_{il}\ {}_{lj}\ {}_{ij}\ {}_{il}\ {}_{lj}}{{}_{j<l}\ {}_{ij}\ {}_{il}\ {}_{ij}\ {}_{il}}$	Weighted Cliquishness or Clustering Coefficient (10).
wPsi_H	$Psi\_H_i = {}_{i}^{H} * {}_{i}$	Weighted Helix-like Psi angle.<br>The delta takes value 1 if the angle is in the range [-77;-17] or 0 otherwise.
wPsi_S	$Psi\_S_i = {}_{i}^{S} * {}_{i}$	Weighted Sheet-like Psi angle. The delta takes value 1 if the angle is in the range [94;154] or 0 otherwise.
wPsi_I	$Psi\_I_i = {}_{i}^{I} * {}_{i}$	Weighted Irregular Psi angle. The delta takes value 1 if the angle is in one of the following ranges: [-180,-77), (-17;94), (154;180] or 0 otherwise.
wPhi_H	$Phi\_H_i = {}_{i}^{H} * {}_{i}$	Weighted Helix like Phi angle. The delta takes value 1 if the angle is in the range [-87;-27] or 0 otherwise.
wPhi_S	$Phi\_S_i = {}_{i}^{S} * {}_{i}$	Weighted Sheet like Phi angle. The delta takes value 1 if the angle is in the range [-159;-99] or 0 otherwise.
wPhi_I	$Phi\_I_i = {}_{i}^{I} * {}_{i}$	Weighted Irregular Phi angle. The delta takes value 1 if the angle is in one of the following ranges: [-180,-159), (-99;-87), (-27;180] or 0 otherwise.
Phi	-	Phi diedral angle
Psi	-	Psi diedral angle
TCD	$wTCD_i = \frac{1}{2N^2}\ {}^{N}_{j\ i}\ ij\ {}^{c}_{ij}$	Total Contact Distance (11).

Table SI-4. Formulae and description of 3D-Thermodynamics Indices.

Acronym	Formula	Description
$Gc_{(F)}$	$G_c(F)_i = RT(N \quad 1)p_i \ln p_i$,<br>$p_i = {\frac{3}{2 \quad (i \quad 1)3.8^2}}^{3/2} \quad e^{\frac{3r_i^2}{2(i \quad 1)3.8^2}}$	Configurational free energy of a folded state. Index based on a “random-flight” model of the protein chain. (1)<br>Where r_i represents the distance to the first residue in the chain.
$W_{(F)}$	$W_i^F = {}_{j=1}^{N} \quad {}_{ij}^{ng} \quad {}_{j}^{s} N_j^w$	Number of water molecules close to a residue in a folded state.(2,3)<br>Where δ^{ng} takes value 1 if the pair of residues are neighbours, using a cutoff for the spatial distance (9.4 Å), or 0 otherwise. In the same way δ^s takes value 1 if the residue is superficial, using a cutoff for the solvent accessible surface area, or 0 otherwise. The parameters N^w represents the number of associated water molecules to the sidechain of a residue (4).
$Gw_{(F)}$	$G_w(F)_i = \quad TR \quad {}_{hyd} \ln \frac{W_i^F!}{(W_i^F \quad N_i^w)!}$	Free energy contribution of the entropy of the first shell of water molecules in a folded state (3). δ_{hyd} takes value 1 if the residue has non-zero N_i^w, or zero otherwise.
$Gs_{(F)}$	$G_s(F)_i = H_i A_i^F$	Interfacial free energy contribution of a folded state. Where H_i is hydrophobicity in Kyte-Doolittle scale (5) and A^F is the solvent accessible surface area of a residue in a folded state.
ΔG_s	$G_{s\,i} = G_s(F)_i \quad G_s(U)_i$	Interfacial free energy variation.
HBd	$Hbd_i = 0.5 \; {}_{j=1}^{N} \; ({}_{ij}^{N} + {}_{ij}^{O})$<br>Geometric definition of a H-bond:<br>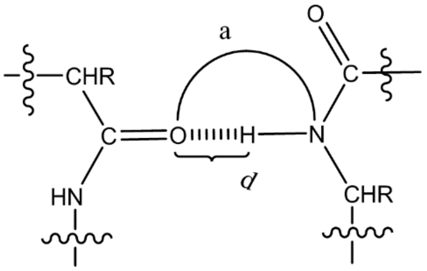<br>d < 2.5 Å<br>a = 120º -- 180º	Number of backbone’s hydrogen bonds.<br>Where ${}_{ij}^{N}$ takes value 1 if the Nitrogen atom of residue i is H-bonded with the Oxygen atom of residue j and 0 otherwise. In the same way ${}_{ij}^{O}$ takes value one if the Oxygen atom of residue i is H-bonded with the Nitrogen atom of residue j and zero otherwise.

ΔG_{el}	$G_{el\,i} = \quad \frac{k_{el}}{2\bar{r}^2} {}_{j=1}^{N} \frac{q_i q_j r_i r_j}{r_{ij}}$	Free energy contribution of the charge distribution within the protein. The parameters q are the Electronic Charge Indices of each residue (6). Parameter k_{el}= 7.608.
ΔG_{w}	$G_{w\,i} = k_w (G_w(F)_i \quad G_w(U)_i)$	Folding free energy contribution of the entropy of the first shell of water molecules.
ΔG_{LJ}	$G_{LJ\,i} = \frac{k_{LJ}}{2} {}_{j=1;\ \lvert j \quad i \rvert > 1}^{N} \quad \frac{3.965}{r_{ij}}^{12} \quad \frac{3.965}{r_{ij}}^{6}$	Residue-level Lennard-Jones interactions. Parameter k_{LJ} = 63.981.
ΔG_{tor}	$G_{tor\,i} = k_{tor}[(\cos^2 2 \quad {}_i \quad 1) + 0.256(\cos^2 2 \quad {}_i \quad 1)]$	Free energy contribution of backbone torsion angles. Parameter k_{tor}= 1.219.

Table SI-5. Summary of the definitions of amino acid groups.

Acronym	Included Residues	Description
**AHR**	ALA, CYS, GLN, GLU, HIS, LEU, LYS, MET	Common residues in alpha helix motifs.
**BSR**	ILE, PHE, THR, TRP, TYR, VAL	Common residues in beta sheet motifs.
**RTR**	ASN, ASP, GLY, PRO, SER	Common residues in reverse turn motifs.
**PCR**	ARG, HIS, LYS	Positive-charged residues.
**NCR**	ASP, GLU	Negative-charged residues.
**UCR**	ASN, CYS, GLN, SER, THR, TYR	Polar non-charged residues.
**ARM**	HIS, PHE, TRP, TYR	Aromatic residues.
**ALR**	ALA, GLY, ILE, LEU, MET, PRO, VAL	Aliphatic residues.
**UFR**	GLY, PRO	Common unfolding-promoter residues
**NPR**	ALA, GLY, ILE, LEU, MET, PHE, PRO, TRP, VAL	Non-polar residues.
**PLR**	ARG, ASN, ASP, CYS, GLN, GLU, HIS, LYS, SER, THR, TYR	Polar residues.

Table SI-6. Aggregation operators: p-norms.

**Acronym**	**Formula**	**Description**
N1	$N1 = \sum_{i=1}^{N} \left\|L_i\right\|$	Minkowski's norms (p = 1) Manhattan norm. Where L_i represents each index of the group of indices and N the number of indices in the group.
N2	$N2 = \sqrt{\sum_{i=1}^{N} \left\|L_i\right\|^2}$	Minkowski's norms (p = 2) Euclidean norm. Where L_i represents each index of the group of indices and N the number of indices in the group.

Table SI-7. Aggregation operators: Central tendency measues (First Statistical Moment).

Acronym	Formula	Description
G	$G = \sqrt[N]{\overset{N}{\underset{i=1}{}} L_i}$	Geometric Mean.<br>Where N is the number of indices in the group.
M	$m_\alpha = \frac{L_1^\alpha + L_2^\alpha + \ldots + L_N^\alpha}{N}^{\frac{1}{\alpha}}$	Arithmetic Mean<br>(potencial with α = 1)
P2		Quadratic Mean<br>(potencial with α = 2)
A		Harmonic Mean<br>(potential with α = -1)

Table SI-8 Aggregation operators: Dispersion and distribution measures (Highest Statistical Moments).

Acronym	Formula	Description
V	$V = \frac{{}_{i=1}^{N}\left(L_i \quad \bar{L}\right)^2}{N - 1}$	Variance.<br>Where N is the number of indices in the group.
S	$S = \frac{N(X_3)}{(N \quad 1)(N \quad 2)(DE)^3}$<br>$X_3 = {}_{a=1}^{N}(L_a \quad \bar{L})^3$	Skewness.<br>Where N is the number of indices in the group and $(DE)^3$ is the standard deviation raised to the 3rd power
K	$k = \frac{N(N+1)X_4 \quad 3(X_2)(X_2)(N \quad 1)}{(N \quad 1)(N \quad 2)(N \quad 3)(DE)^4}$<br>$X_j = {}_{a=1}^{N}(L_a \quad \bar{L})^j$	Kurtosis.<br>Where $(DE)^4$ is the standard deviation raised to the fourth power
CV	$c_v = {}^{s}\!/_{\bar{L}}$	Variation Coefficient
Q1	$P25 = \frac{N}{4} + \frac{1}{2}$	Percentile 25.<br>Where N is the number of indices in the group.
Q2	$P50 = \frac{N}{2} + \frac{1}{2}$	Percentile 50.<br>Where N is the number of indices in the group.
Q3	$P75 = \frac{3N}{4} + \frac{1}{2}$	Percentile 75.<br>Where N is the number of indices in the group.
I50	I50 = P75 P25	Inter-quartileRange

Table SI-9. Aggregation operators: Information-Theory-based measures.

Acronym	Formula (Equation)	Description
MI	$MI = \sum_{i=1}^{K} \frac{N_k}{N} \log_2 \frac{N_k}{N}$	Mean Information Content.<br>Where N_k is the number of indices in the same bin, K is the number of bins defined to compute the operator and N is the total number of indices in the group.
TI	$TI = N \log_2 N \quad \sum_{k=1}^{K} N_k \log_2 N_k$	Total Information Content.
SI	$SI = \frac{TI}{N \log_2 N}$	Standarized Infomation Content

Table SI-10. Structural diversity summary of the D&D enzyme subset according to SCOP hierarchal database.

Class	Folds	Superfamily/family
**Alpha and beta proteins a/b (372)**	TIM beta/alpha-barrel (80)	**1.** Trans-glycosidases (27)<br>**2.** FMN-linked Oxidoreductases (8)<br>**3.** Phosphoenolpyruvate/pyruvate (4)<br>**4.** Ribulose-phoshate binding (4)<br>**5.** Other (41)
	NAD(P)-binding Rossmann-fold domains (38)	**1.** Tyrosine-dependent oxidoreductase (13)<br>**2.** Glyceraldehyde-3-phosphatase (6)<br>**3.** Aminoacid dehydrogenase-l (5)<br>**4.** 6-phosphogluconate dehydrogenase (5)<br>**5.** LDH N-terminal domain-like (4)<br>**6.** Lactate & malate dehydrogenase (4)<br>**7.** Aminoacid dehydrogenases (3)<br>**8.** Other (18)
	P-loop containing nucleoside triphosphate hydrolase (22)	**1.** Nucleotide and nucleoside kinases (8)<br>**2.** Nitrogenase iron protein (5)<br>**3.** RecA protein-like (ATPase domain (3)<br>**4.** G proteins (2)<br>**5.** Microbial and mitochondrial ADK (2)<br>**6.** F1 ATP synthase (2)<br>**7.** Other (6)
	alpha/beta-Hidrolases (18)	**1.** Acetylcholinesterase-like (3)<br>**2.** Fungal lipases (2<br>**3.** Cutinase-like (2)<br>**4.** Haloalkane dehalogenase (2)<br>**5.** Bacterial lipase (2)<br>**6.** Porlyl_oligopeptidase (1)<br>**7.** Haloperoxidase (1)<br>**8.** Other (6)
	Flavodoxin-like a/b/a parallel beta-sheet (17)	**1.** Class I glutamine amidotransferase like (4)<br>**2.** NAD(P)-binding Rossmann-fold domains (3)<br>**3.** Flavoproteins ((3)<br>**4.** Cobalamin (vitamin B12)-binding domain (2)<br>**5.** Formate/glycerate dehydrogenase (2)<br>**6.** Glutathione synthetase ATP-binding domain (2)<br>**7.** Cobalamin (vitamin B12)-dependent enzymes (2)<br>**8.** Other (10)
	PLP-dependent transferase-like (16)	**1.** GABA-aminotransferase-like (6)<br>**2.** Cystathionine synthase-like (5)<br>**3.** AAT-like (4)<br>**4.** Beta-eliminating lyases (1)
	Ribonuclease H-like motif-- a/b/a; mixed beta-sheet (14)	**1.** Ribonuclease H-like (8)<br>**2.** Actin-like ATPase domain (4)<br>**3.** DNA/RNA polymerases (3)<br>**4.** Creatinase/prolidase (1)<br>**5.** Methyl transferases... (2)<br>**6.** Creatinase/aminopeptidase (1)
	Others (240)	**1.** TIM beta/alpha-barrel (29)<br>**2.** NAD(P)-binding Rossmann-fold (23)<br>**3.** FAD/NAD(P)-binding domain (13)<br>**4.** Nucleotide-diphospho-sugar tranferase (11)<br>**5.** Thioredoxin fold (10)<br>**6.** Phosphorylase/hydrolase-like (10)<br>**7.** S-adenosyl-L-methionine-methyltransferase (9)<br>**8.** Other (214)
**Alpha and beta proteins (a+b) (210)**	Protein kinase-like (PK-like), two alpha+beta domains (17)	**1.** Protein kinases (14)<br>**2.** SH3-domain (2)<br>**3.** SH2 domain (Pfam 00017) (2)<br>**4.** Ras-binding domain (1)<br>**5.** Phoshoinositide 3-kinase (1)<br>**6.** MHCK/EF2 kinase (1)<br>**7.** APH phosphotransferases (1)

		**8.** Other (1)
	Ferredoxin-like (alpha+beta sandwich with antiparallel beta-sheet) (16)	**1.** RuBisCo, C-terminal domain (2)<br>**2.** 4Fe-4S ferredoxins (2)<br>**3.** RuBisCO, N-terminal domain (2)<br>**4.** Nitrite/Sulfite reductase (1)<br>**5.** Anticodon-binding domain of PheRS (1)<br>**6.** GHMP Kinase (1)<br>**7.** Nucleic acid-binding protein (1)<br>**8.** Other (15)
	TIM beta/alpha-barrel (15)	**1.** Enolase C-terminal domain (4)<br>**2.** (Trans)glycosidases (4)<br>**3.** Enolase N-terminal domain (4)<br>**4.** RuBisCo, C-terminal domain (2)<br>**5.** beta-N-acetylhexosaminida (2)<br>**6.** FMN-linked oxidoreductase (2)<br>**7.** E set domains (2)<br>**8.** Other (10)
	NAD(P)-binding Rossmann-fold (11)	**1.** G-3-P dehydrogenase (6)<br>**2.** LDH N-terminal domain-like (4)<br>**3.** Lactate & malate dehydrogenase (4)<br>**4.** Dihydrodipicolinate reductase (2)<br>**5.** GAPDH-like (2)<br>**6.** Succinyl-CoA synthetase (1)<br>**7.** Glucose 6-phosphate dehydrogenase (1)<br>**8.** Other (2)
	Class II aaRS and biotin synthetases (large mixed beta-sheet) (10)	**1.** Class II aminoacyl-tRNA synthetase (3)<br>**2.** Anticodon-binding domain (6)<br>**3.** Seryl-tRNA synthetase (1)<br>**4.** GAD domain (1)<br>**5.** Myf domain (1)<br>**6.** Biotin repressor-like (1)<br>**7.** Other (3)
	FAD/NAD(P)-binding domain - central parallel beta-sheet (10)	**1.** FAD/NAD-linked reductases N-ter (4)<br>**2.** FAD/NAD-linked reductases C-ter (4)<br>**3.** FAD-linked reductases, N-terminal (3)<br>**4.** Succinate dehydrogenase/fumarate (4)<br>**5.** Ferredoxin domains (1)<br>**6.** Dihydropyrimidine dehydrogenase.. (1)<br>**7.** Other (6)
	Zincin-like (mixed beta sheet) (9)	**1.** Metalloproteases (7)<br>**2.** (Trans)glycosidases (2)<br>**3.** beta-N-acetylhexosaminida (2)<br>**4.** E set domains (1)<br>**5.** beta-Roll (superhelix turn) (1)<br>**6.** Carbohydrate-binding domain (1)
	Others (183)	**1.** TIM beta/alpha-barrel (13)<br>**2.** NAD(P)-binding Rossmann-fold (11)<br>**3.** Ferredoxin-like (alpha+beta) (11)<br>**4.** FAD/NAD(P)-binding domain (10)<br>**5.** Class II aaRS and biotin synthetses (9)<br>**6.** ATP-grasp (9)<br>**7.** Lysozyme-like (8)<br>**8.** Other (176)
**All beta proteins (144)**	TIM beta/alpha-barrel (16)	**1.** (Trans)glycosidases (9)<br>**2.** Glycosyl hydrolase domain (6)<br>**3.** E set domains (3)<br>**4.** Phosphoenolpyruvate domain. (2)<br>**5.** Alanine racemase C-terminal. (2)<br>**6.** Chitinase insertion domain (2)<br>**7.** PLP-binding barrel (2)<br>**8.** Other (8)
	Trypsin-like serine proteases (barrel) (14)	**1.** Eukaryotic proteases (9)<br>**2.** Prokaryotic proteases (3)<br>**3.** Viral cysteine protease (1)<br>**4.** EGF-type module (1)<br>**5.** Viral proteases (beta sheet) (1)
	Immunoglobulin-like beta sándwich	**1.** E set domains (6)<br>**2.** (Trans)glycosidases (3)<br>**3.** Galactose-binding domain (2)

		**4.** Cu,Zn superoxide dismutase (2)<br>**5.** CBD9-like (1)<br>**6.** Immunoglobulin (1)<br>**7.** Galactose oxidase, centra ... (1)<br>**8.** Other (6)
	Acid proteases- barrel closed (9)	**1.** Pepsin-like (5)<br>**2.** Retroviral protease (4)
	OB-fold closed or partially open (9)	**1.** Nucleic acid-binding prot ... (6)<br>**2.** Class II aaRS and biotin ... (4)<br>**3.** Inorganic pyrophosphatase ... (2)<br>**4.** GAD domain-like (Superfa ... (1)<br>**5.** Anticodon-binding domain ... (1)<br>**6.** DNA ligase/mRNA capping e ... (1)<br>**7.** Staphylococcal nuclease (... (1)<br>**8.** Other (2)
	6-bladed beta-propeller (8)	**1.** Sialidases (6)<br>**2.** E set domains (1)<br>**3.** Galactose-binding domain (1)<br>**4.** Soluble quinoprotein glucos dehydrogenase (1)<br>**5.** Concanavalin A-like lectine (1)<br>**6.** Thermostable phytase (1)
	SH3-like barrel (8)	**1.** SH3-domain (Superfamily) (4)<br>**2.** Protein kinase-like (2)<br>**3.** Electron transport accessesory proteins (2)<br>**4.** SH2 domain (Superfamily) (2)<br>**5.** Nitrile hydratase alpha chains (1)<br>**6.** Fumarylacetoacetate hydrolase (1)<br>**7.** Class II aaRS and biotin synthetases (1)<br>**8.** Other (2)
	Others (105)	**1.** TIM beta/alpha-barrel (16)<br>**2.** Barrel-sandwich hybrid (6)<br>**3.** Double-stranded beta-helix (6)<br>**4.** Glycosyl hydrolase domain (6)<br>**5.** Immunoglobulin-like beta-sandwich (6)<br>**6.** Concanavalin A-like lectins (6)<br>**7.** OB-fold (barrel, closed) (6)<br>**8.** Other (87)
**All alpha proteins (99)**	alpha/alpha toroid (8)	**1.** Six-hairpin glycosidases (3)<br>**2.** Terpenoid cyclases/Protein (3)<br>**3.** Hyaluronate lyase-like (1)<br>**4.** Chondroitin AC/alginate (1)<br>**5.** Seven-hairpin glycosidase (1)<br>**6.** Protein prenylyltransferase (1)<br>**7.** Galactose mutarotase-like (1)<br>**8.** Other (2)
	Thioredoxin fold (7)	**1.** Class alpha GST (2)<br>**2.** Class alpha GST (2)<br>**3.** Class zeta GST (1)<br>**4.** Class sigma GST (1)<br>**5.** Class phi GST (1)<br>**6.** Class pi GST (1)<br>**7.** Class sigma GST (1)<br>**8.** Other (4)
	GST C-terminal domain-like (7)	**1.** Class alpha GST (2)<br>**2.** Class alpha GST (2)<br>**3.** Class zeta GST (1)<br>**4.** Class sigma GST (1)<br>**5.** Class phi GST (1)<br>**6.** Class pi GST (1)<br>**7.** Class sigma GST (1)
	DNA/RNA-binding 3-helical bundle (7)	**1.** \`Winged helix\` DNA-binding (5)<br>**2.** Creatinase/aminopeptidase (2)<br>**3.** Methylated DNA-protein cyteine methyltransferase (1)<br>**4.** Class II aaRS and biotin synthetase (1)<br>**5.** C-ter domain of transcriptional repressors (1)

		**6.** Nucleic acid-binding protein (1)<br>**7.** Restriction endonuclease (1)<br>**8.** Other (3)
	Cytochrome P450 (multihelical) (6)	**1.** Cytochrome P450-CAM (1)<br>**2.** Cytochrome P450-NOR (1)<br>**3.** Cytochrome P450 bm-3 (1)<br>**4.** Cytochrome P450-ERYF (1)<br>**5.** Cyp119 (thermophilic P450) (1)<br>**6.** Cytochrome p450 14 alpha-demethylase. (1)
	NAD(P)-binding Rossmann-fold domains (5)	**1.** L-3-hydroxyacyl CoA dehydrogenase (2)<br>**2.** L-3-hydroxyacyl CoA dehydrogenase (2).<br>**3.** UDP-glucose dehydrogenase (1)<br>**4.** 6-phosphogluconate dehydrogenase (1)<br>**5.** UDP-glucose dehydrogenase (2)<br>**6.** Class II ketol-acid reductase (1)<br>**7.** Other (2)
	6-phosphogluconate dehydrogenase C-terminal domain-like (5)	**1.** L-3-hydroxyacyl CoA dehydrogenase (2)<br>**2.** L-3-hydroxyacyl CoA dehydrogenase (2<br>**3.** UDP-glucose dehydrogenase (1)<br>**4.** 6-phosphogluconate dehydrogenase (1)<br>**5.** UDP-glucose dehydrogenase (1)<br>**6.** UDP-glucose dehydrogenase (1)<br>**7.** Class II ketol-acid reductase (1)<br>**8.** Other (2)
	Others (78)	**1.** DNA/RNA-binding 3-helical bundle (7)<br>**2.** SAM domain-like (4)<br>**3.** alpha-alpha superhelix (4)<br>**4.** Ferritin-like (4)<br>**5.** Ferredoxin-like (alpha+beta) (4)<br>**6.** alpha/alpha toroid (4)<br>**7.** L-aspartase-like (3)<br>**8.** Other (71)
**Multi-domain proteins (alpha + beta) (26)**	beta-lactamase/transpeptidase-like (6)	**1.** beta-Lactamase, class A (2)<br>**2.** D-ala carboxy/transpetidase (2)<br>**3.** Class D beta-lactamase (1)<br>**4.** AMPC beta-Lactamase, class C (1)
	Carbohydrate phosphatase-alpha+beta N-terminal domain (5)	**1.** Fructose-1,6-bisphosphatase (3)<br>**2.** Inositol monophosphatase (1)<br>**3.** Archaeal inositol monophosphatase (1)
	DNA/RNA polymerases (5)	**1.** DNA polymerase I (2)<br>**2.** DnaQ-like 3'-5' exonuclease (2)<br>**3.** dsRNA phage RNA-dependent (1)<br>**4.** RNA-dependent RNA-polymerase (1)<br>**5.** Reverse transcriptase (1)<br>**6.** Ribonuclease H (1)
	Ribonuclease H-like motif (3)	**1.** DNA polymerase I (2)<br>**2.** DnaQ-like 3'-5' exonuclease (2)<br>**3.** Reverse transcriptase (1)<br>**4.** Ribonuclease H (1)
	D-aminoacid aminotransferase-like PLP-dependent enzymes (2)	**1.** Branched-chain aa amino-transferase (1)<br>**2.** D-aa aminotransferase (1)
	Heme-dependent catalase-like (2)	**1.** Catalase II (1)<br>**2.** Catalase I (1)<br>**3.** Catalase, C-terminal domain (1)
	Acyl-CoA dehydrogenase C-terminal domain- like (1)	**1.** Acyl-CoA dehydrogenase C-terminal domain- like
	Others (7)	**1.** Dehydroquinate synthase-l (1)<br>**2.** HydB/Nqo4-like domain (1)<br>**3.** DNA primase core (1)<br>**4.** Acyl-CoA dehydrogenase (1)

		5. Acetyl-CoA synthetase-like (1)<br>6. Prokaryotic type I DNA topoisomerase (1)<br>7. Bromodomain-like (1)<br>8. Other (2)
**Small proteins (11)**	Rubredoxin-like (4)	1. Microbial and mitocondrial ADK (2)<br>2. P-loop containing nucleoside triphospate hydrolases (2)<br>3. Zinc beta-ribbon (1)<br>4. Nucleotidylyl transferase (1)<br>5. Anticodon-binding domain (1)<br>6. Methionyl-tRNA synthetase (1)
	Kringle-like (disulfide-rich fold; nearly all-beta) (3)	1. Plasminogen (2)<br>2. Prothrombin (1)<br>3. Prothrombin (1)
	Knottins (small inhibitors, toxins, lectins) (2)	1. Bowman-Birk inhibitor (1)<br>2. EGF/Laminin (Superfamily) (1)<br>3. Trypsin-like serine protease (1)
	P-loop containing nucleoside triphosphate hydrolases (2)	1. Microbial and mitocondrial ADK (2)<br>2. Adenylate kinase (2)
	GLA-domain (1)	1. GLA-domain (1)
	7-bladed beta-propeller (1)	1. Methylamine dehydrogenase
	Cysteine-rich domain (1)	1. Cysteine-rich domain (1)
	Others (3)	1. 7-bladed beta-propeller (1)<br>2. Trypsin-like serine protease (1)<br>3. Adenine nucleotide alpha hydrolase (1)<br>4. Methylamine dehydrogenase (1)<br>5. Knottins (1)<br>6. Anticodon-binding domain AARS class I (1)<br>7. Rubredoxin-like bound fold (1)
**Membrane and cell surface proteins and peptides (2)**	Heme-binding four-helical bundle (1)	1. alpha-helical ferredoxin
	Spectrin repeat-like (1)	1. Succinate dehydrogenase/fumarate reductase flavoprotein C- terminal domain
	Transmembrane beta-barrel (1)	1. Outer membrane phospholipase A (OMPLA)
	Succinate dehydrogenase/fumarate reductase flavoprotein C- terminal domain (1)	1. Succinate dehydrogenase/fumarate reductase flavoprotein, catalytic domain
	beta-Grasp (ubiquitin-like (1)	1. 2Fe-2S ferredoxin-like
	Globin-like (1)	2. alpha-helical ferredoxin

Table SI-11. Structural diversity summary of the D&D non-enzyme subset according to SCOP hierarchal database

Class	Folds	Superfamily/family
**Alpha and beta proteins a/b (74)**	P-loop containing nucleoside triphosphate hydrolases (21)	**1.** G proteins (core: mixed beta sheet) (12)<br>**2.** Elongation factors (4)<br>**3.** Nitrogenase iron protein-like (3)<br>**4.** EF-Tu/eEF-1alpha/eIF2-gamma Cterminal domain (3)<br>**5.** Extended AAA-ATPase domain (2)<br>**6.** Motor proteins (2)<br>**7.** Domain of the SRP/SRP receptor G proteins (2)<br>**8.** Other (9)
	Periplasmic binding protein-like II (16)	**1.** Phosphate binding protein-like (11)<br>**2.** Transferrin (further duplication) (5)
	Flavodoxin-like (6)	**1.** CheY-like (Superfamily) (4)<br>**2.** Flavoproteins (Superfamily) (2)<br>**3.** C-terminal effector domain (1)
	Thioredoxin fold (5)	**1.** Thioltransferase (3)<br>**2.** Glutathione S-transferase Nterminal (1)<br>**3.** Glutathione peroxidase-like (1)<br>**4.** Glutathione S-transferase Cterminal (1)
	Periplasmic binding protein-like I (5)	**1.** D-allose-binding protein (1)<br>**2.** Galactose/glucose-binding protein (1)<br>**3.** Leucine-isoleucine-,valine bonding protein (1)<br>**4.** D-ribose-binding protein (1)<br>**5.** L-arabinose-binding protein (1)
	vWA-like (4)	**1.** Integrin alpha2-beta1 (2)<br>**2.** Integrin alpha M (CR3, CD11b/CD18 (1)<br>**3.** Collagen-like peptides (1)<br>**4.** Integrin CD11a/CD18 (LFA1) (1)
	Reductase/isomerase/elongation factor common domain (4)	**1.** Elongation factor Tu (EF-Tu domain 2 (2)<br>**2.** Elongation factor Tu (EF-Tu) (2)<br>**3.** Elongation factor Tu (EF-Tu Nterminal (2)<br>**4.** Elongation factor eEF-1alpha domain (1)<br>**5.** Initiation factor IF2/eIF (1)<br>**6.** Guanine nucleotide Exchange factor domain (1)<br>**7.** Elongation factor eEF-1alpha domain2 (1)<br>**8.** Other (2)
	Others (31)	**1.** P-loop containing nucleoside thriphosphate hydrolase (11)<br>**2.** Reductase/isomerase/elongation factor common domain (4)<br>**3.** TIM beta/alpha-barrel (3)<br>**4.** Elongation factor/aminomethyltransferase common domain (3)<br>**5.** Four-helical up-and-down bunle (2)<br>**6.** Transducin (alpha subunit) insertion domain (2)<br>**7.** Leucine-rich repeat, LRR (2)<br>**8.** Other (21)
**Alpha and beta proteins (a+b) (96)**	beta-Grasp (ubiquitin-like) (13)	**1.** Superantigen toxins, C-terminal domain (5)<br>**2.** Bacterial enterotoxins (5)<br>**3.** 2Fe-2S ferredoxin-like (superfamily) (3)<br>**4.** Immunoglobulin-binding domains (3)<br>**5.** Ubiquitin-like (Superfamily) (1)

		**6.** Staphylokinase/streptokinase (superfamily) (1)
	Ferredoxin-like (10)	**1.** 4Fe-4S ferredoxins (Superfamily) (3)<br>**2.** Viral DNA-binding domains (2)<br>**3.** Molybdenum cofactor biosythesis protein C(1)<br>**4.** eEF-1beta-like (Superfamily) (1)<br>**5.** EF-Tu/eEF-1alpha/eIF2-gamma Cterminal domain (1)<br>**6.** RNA-binding domain, RBD (1)<br>**7.** P-loop containing nucleoside triphosphate hydrolases (1)<br>**8.** Other (3)
	C-type lectin-like (9)	**1.** C-type lectin domain (8)<br>**2.** Triple coiled coil domain of C type lectines (1)<br>**3.** Endostatin (decorated with many insertions in the common fold (1)
	IL8-like (6)	**1.** Chemokine domain of fractaline (1)<br>**2.** Stromal cell-derived factor 1 (1)<br>**3.** Platelet factor 4, PF4 (1)<br>**4.** RANTES (regulated upon activation T cell expressed and secretes (1)<br>**5.** Interleukin-8, IL-8 (1)<br>**6.** Monocyte chemoattractant protein (1)
	OB-fold (5)	**1.** Streptococcal superantigen Smez-2 (1)<br>**2.** Streptococcal superantigen SSA (1)<br>**3.** Staphylococcal enterotoxin B (1)<br>**4.** Toxic shock syndrome toxin-1 (1)<br>**5.** Staphylococcal enterotoxin B (1)<br>**6.** Toxic shock syndrome toxin 1 (1)<br>**7.** Streptococcal superantigen SSA (1)<br>**8.** Other (2)
	Profilin-like (5)	**1.** PYP-like sensor domain (2)<br>**2.** Profilin (actin-binding protein) (2)<br>**3.** GAF domain-like (1)
	SH2-like (4)	**1.** Growth factor receptor-bound protein 2 (2)<br>**2.** N-terminal domain of cbl ... (1)<br>**3.** Cbl (1)<br>**4.** Cbl (1)<br>**5.** Phosphatidylinositol 3-kinase (1)
	Others (52)	**1.** Cystatin-like (Core: alpha beta, helix pack against coiled antiparallell beta sheet... (4)<br>**2.** TBP-like (beta-alpha-beta) (3)<br>**3.** CI-2 family of serine protease inhibitors (2)<br>**4.** HIT-like (alpha-beta(3)- ... (2)<br>**5.** Cytochrome b5-like heme/steroid binding domain) (2)<br>**6.** DNA clamp (contains two helices and two beta sheets (2)<br>**7.** Immunoglobulin-like beta- sandwich (2)<br>**8.** Other (37)
**All beta proteins (156)**	Immunoglobulin-like beta-sandwich (37)	**1.** Immunoglobulin (Superfamily) (23)<br>**2.** Fibronectin type III (Superfamily) (6)<br>**3.** E set domains (\`Early\` Ig likes fold families(5)<br>**4.** 4-helical cytokines (2)<br>**5.** Hemocyanin, N-terminal domain (2)<br>**6.** Di-copper centre-containing domain (2)<br>**7.** Actinoxanthin-like (Superfamily) (1)<br>**8.** Other (3)
	OB-fold (18)	**1.** Nucleic acid-binding proteins ... (8)<br>**2.** Bacterial enterotoxins (7)<br>**3.** Superantigen toxins, C-terminal domain (5)

		**4.** MOP-like (Superfamily) (2)<br>**5.** RuvA domain 2-like (1)<br>**6.** DNA helicase RuvA subunit, C terminal domain (1)<br>**7.** TIMP-like (Superfamily) (1)<br>**8.** Other (3)
	Lipocalins (11)	**1.** Retinol binding protein-like (9)<br>**2.** Fatty acid binding protein-like (2)
	Cupredoxin-like (8)	**1.** Plastocyanin/azurin-like mono domain protein (7)<br>**2.** Nitrosocyanin (1)
	SH3-like barrel (7)	**1.** SH3-domain (Superfamily) (3)<br>**2.** Cell growth inhibitor/plasmid maintenance toxic components (1)<br>**3.** Chromo domain-like (SH3-like barrel (1)<br>**4.** Myosin S1 fragment, N-terminal domain (1)<br>**5.** Translation proteins SH3-like domain (1)<br>**6.** P-loop containing nucleoside triphosphate hydrolases (1)<br>**7.** Nucleic acid-binding proteins (1)
	beta-Trefoil (7)	**1.** Cytokine (Superfamily) (3)<br>**2.** STI-like (Superfamily) (2)<br>**3.** Agglutinin (superfamily) ... (1)<br>**4.** Ricin B-like lectins (Superfamily) (1)
	Concanavalin A-like lectins/glucanases (6)	**1.** Legume lectins (3)<br>**2.** Galectin (animal S-lectin) (2)<br>**3.** Pentraxin (pentaxin) (1)
	others (78)	**1.** OB-fold (barrel, closed or partly opened<br>**2.** Immunoglobulin-like beta sandwich (6)<br>**3.** Common fold of diphtheria toxin (6)<br>**4.** Double-stranded beta-helix (5)<br>**5.** beta-Grasp (ubiquitin-like) (5)<br>**6.** P-loop containing nucleoside triphospahte hydrolases (5)<br>**7.** Reductase/isomerase/elongation factor common domain (4)<br>**8.** Other (65)
**All alpha proteins (132)**	EF Hand-like (15)	**1.** Calmodulin-like (Duplication) (8)<br>**2.** Parvalbumin (6-helices; arrays 3 hairpirins) (2)<br>**3.** S100 proteins (dimer) (2)<br>**4.** EF-hand modules in multidomain proteins (2)<br>**5.** WW domain (1)<br>**6.** N-terminal domain of cbl (1)<br>**7.** Calbindin D9K (1)<br>**8.** Other (1)
	Four-helical up-and-down bundle (11)	**1.** Apolipoprotein (Superfamily) (3)<br>**2.** P-loop containing nucleoside triphosphate hydrolases (2)<br>**3.** Aspartate receptor, ligand-binding domain (2)<br>**4.** Domain of the SRP/SRP receptor G proteins (2)<br>**5.** FKBP12-rapamycin-binding domain (1)<br>**6.** TMV-like viral coat proteins (1)<br>**7.** Cytochromes (Heme-containing proteins (1)<br>**8.** Other (1)
	DNA/RNA-binding 3-helical bundle (11)	**1.** \`Winged helix\` DNA-binding domain (6)<br>**2.** Homeodomain-like (consists only of helices) (2)<br>**3.** C-terminal effector domain of the bipartite response regulators (2)<br>**4.** Iron-dependent repressor protein (1)<br>**5.** Tetracyclin repressor-like C terminal domain (1)<br>**6.** MOP-like (Superfamily) (1)

		**7.** P-loop containing nucleoside triphosphate hydrolases (1)<br>**8.** Other (4)
	Globin-like (9)	**1.** Globins (Heme-binding protein (7)<br>**2.** Phycocyanin-like phycobilisome proteins (2)
	Cytochrome c (8)	**1.** Cytochrome c2 (3)<br>**2.** Cytochrome c6 (2)<br>**3.** Cytochrome c551 (1)<br>**4.** Cytochrome c552 (1)<br>**5.** Photosystem II associated Cyt c549 (1)
	alpha-alpha superhelix	**1.** ARM repeat (Superfamily) (3)<br>**2.** TPR-like (Superfamily) (2)<br>**3.** ENTH/VHS domain (Superfamily) (2)<br>**4.** GAT-like domain (Superfamily) (1)<br>**5.** 14-3-3 protein (Superfamily) (1)<br>**6.** beta-Catenine bound non-globular protein regions (1)
	4-helical cytokines	**1.** Long-chain cytokines (3)<br>**2.** Interferons/interleukin-10 (3)<br>**3.** Short-chain cytokines (2)<br>**4.** Fibronectin type III (2)
	Other (77)	**1.** Nuclear receptor ligand-binding domain (8)<br>**2.** DNA/RNA-binding 3-helical bundle (7)<br>**3.** Multiheme cytochromes (6)<br>**4.** P-loop containing nucleoside triphosphate hydrolases (6)<br>**5.** Immunoglobulin-like beta- sandwich (4)<br>**6.** SAM domain-like (4-5 helices) (3)<br>**7.** OB-fold (barrel, closed or partly opened (3)<br>**8.** Other (59)
**Small proteins (38)**	Knottins (small inhibitors, toxins, lectins) (8)	**1.** Plant lectins/antimicrobial peptides (2)<br>**2.** Scorpion toxin-like (Superfamily) (2)<br>**3.** Leech antihemostatic proteins (2)<br>**4.** EGF/Laminin (Superfamily) (2)
	Cystine-knot cytokines (4)	**1.** Neurotrophin (2)<br>**2.** Transforming growth factor (1)<br>**3.** Platelet-derived growth factor like (1)
	Snake toxin-like (4)	**1.** Erabutoxin A (1)<br>**2.** Fasciculin (1)<br>**3.** Neurotoxin I (1)<br>**4.** Cardiotoxin V4II (Toxin III) (1)
	Rubredoxin-like (4)	**1.** Rubredoxin (3)<br>**2.** Desulforedoxin (1)<br>**3.** Ferritin (1)
	BPTI-like (3)	**1.** Pancreatic trypsin inhibitor (3)
	Defensin-like (2)	**1.** Beta-defensin, BD (1)<br>**2.** Defensin HNP-3 (1)
	HIPIP (high potential iron protein) (2)	**1.** HIPIP (high potential iron protein (2)
	Others (small inhibitors, toxins, lectins) (12)	**1.** Kazal-type serine protease inhibitors (2)<br>**2.** Kringle-like (disulfide-rich fold) (2)<br>**3.** Insulin-like (2)<br>**4.** Crambin-like (disulfide- rich alfa beta fold (2)<br>**5.** Trefoil/Plexin domain-like (1)<br>**6.** Ferritin-like (core: 4 h ... (1)<br>**7.** LDL receptor-like module (1)<br>**8.** Other (3)

**Membrane and cell surface proteins and peptides (14)**	Transmembrane beta-barrels (7)	**1.** Porins (Superfamily) (5)<br>**2.** OMPA-like (forms (8,10) (2)
	Toxins' membrane translocation domains (2)	**1.** delta-Endotoxin (insectocide (1)<br>**2.** delta-Endotoxin (insectocide (1)<br>**3.** Galactose-binding domain-like (1)<br>**4.** Diphtheria toxin, C-terminal domain (1)<br>**5.** Diphtheria toxin, middle domain (1)<br>**6.** ADP-ribosylation (Superfamily) (1)
	Family A G protein-coupled receptor-like (2)	**1.** Bacteriorhodopsin (a light driven proton pump (1)<br>**2.** Sensory rhodopsin II (1)
	Diphtheria toxin, C-terminal domain (1)	**1.** Diphtheria toxin, C-terminal domain
	Light-harvesting complex subunits (1)	**1.** Light-harvesting complex subunits
	Bacterial photosystem II reaction centre (1)	**1.** Bacterial photosystem II reaction centre
	PRC-barrel domain (1)	**1.** PRC-barrel domain
	Other (4)	**1.** Toxins (2)<br>**2.** Common fold of diphtheria toxin (1)<br>**3.** Bacterial photosystem II (1)<br>**4.** PRC-barrel domain (1)<br>**5.** Galactose-binding domain- like (1)<br>**6.** Leukocidin-like (subunit fold contains beta sandwich (1)<br>**7.** Single transmembrane helix (1)<br>**8.** Other (2)
Coiled coil proteins (11)	Parallel coiled-coil (7)	**1.** TRAF domain-like (2)<br>**2.** Trimerization domain of TRAF (2)<br>**3.** Inovirus (filamentous phage) major coat protein (1)<br>**4.** Intermediate filament protein ... (1)<br>**5.** C-type lectin-like (Superfamily) ... (1)<br>**6.** Triple coiled coil domain of c type lectin (1)<br>**7.** Fibrinogen coiled-coil and central regions (1)<br>**8.** Other (1)
	Stalk segment of viral fusion proteins (3)	**1.** Retrovius gp41 protease-resistant core (2)<br>**2.** MoMLV p15 core fragment (1)
	TRAF domain-like (2)	**1.** TNF receptor associated factor 2m(2)<br>**2.** TRAF2 (2)
	C-type lectin-like (1)	**1.** C-type lectin-like
	Antiparallel coiled-coil (1)	**1.** Oligomerization domain of hepatitis delta antigen
**Other 14**		

Table SI-12. Structural information of the selected ProtDCal's features from the different families of descriptors (0D, 1D & 3D)

Descriptors	***Associated Information***
0D type:	
ECI_NO_ALR_N2	Amount of **aliphatic residues**, represented with their electron charge indices (polarity)
Pt_NO_ILE_N2	Amount of **Ile** represented by its probability to exits in a turn motif.
Z1_NO_ALA_N1	Amount of **Ala** represented with its hydrophobicity
ECI_NO_NPR_N2	Amount of **non-polar residues** represented with electron charge indices
DHf_NO_NPR_SI50	Mean content of **non-polar residues** represented with its heat of formation
Xi_NO_GLY_N1	Amount of **Gly** represented with its dihedral angles compressibility
HP_NO_UCR_TI50	Mean content of **non-charged-polar-residues** represented with their hydrophobicity
ISA_NO_HIS_N1	Amount of **His** represented with its non-polar area
DHf_NO_ARG_N1	Amount of **Arg** represented by its heat of formation
1D type:	
L1-9_ES_ALR_MI50	Mean content of **aliphatic residues** represented with their bonds compressibility
Z2_ES_ALR_MI50	Mean content of **aliphatic residues** represented with theirs steric characteristics
Z1_ES_ILE_N1	Amount of **Ile** weighted with their hydrophobic properties
HP_ES_NPR_MI50	Mean content of **non-polar residues** represented with their hydrophobicity
Ap_ES_ARM_N2	Amount of **aromatic residues** represented with their polar area
Z2_ES_PHE_N2	Amount of **Phe** weighted with their steric properties
HP_ES_PRT_MI50	Mean hydrophobicity content of **the protein**
Gs(U)_ES_PRT_MI50	Mean superficial free energy of **the protein**
Z3_ES_RTR_MI50	Mean content of **residues favoring reverse turns**, being represented with their polar properties
Z2_ES_PLR_N1	Amount of **polar residues** weighted with their steric properties
Mw_ES_PLR_MI50	Mean content of **polar residues** represented with their molar weight
Z3_ES_ARG_N1	Amount of **Arg** represented with their polar properties
Xi_ES_HIS_N2	Amount of **His** weighted with their dihedral angles compressibility
3D type:	
wPhiH(ECI)_NO_ALR_N1	**Aliphatic residues**, with α-helix-like *phi* angles, represented with their electron charge indices (polarity)
wFLC(IP)_NO_ALR_Ar	Topology of **aliphatic residues** represented with their isoelectric points
wNc(ISA)_NO_ALR_N2	Topology of **aliphatic residues** represented with their non-polar area
wNc(Z2)_NO_ALR_N2	Topology of **aliphatic residues** represented with their steric properties
wCTP(Z1)_NO_LEU_G	Topology of **Leu** residues represented by the hydrophobic properties
wNLC(ECI)_NO_NPR_N2	Topology of **non-polar residues** represented with their electron charge indices
wNLC(IP)_NO_NPR_MI50	Topology of **non-polar residues** represented with their isoelectric points
wPhiS(ISA)_NO_NPR_G	**Non-polar residues**, with β-sheet-like *phi* angles, represented with their non-polar areas
wCTP(IP)_NO_PHE_N2	Topology of **Phe** residues represented with the isoelectric point
wCTP(Z1)_NO_PHE_I50	Topology of **Phe** residues represented by the hydrophobic properties
wCTP(ISA)_NO_INT10.0_N1	Topology of **internal residues** represented with their non-polar areas
wLCO(IP)_NO_INT10.0_N1	Topology of **internal residues** represented with their isoelectric points
wPhiH(ISA)_NO_INT10.0_G	**Internal residues**, with α-helix-like *phi* angles, represented with their non-polar areas
wPhiI(Z1)_NO_ALR_N2	**Aliphatic residues**, with irregular *phi* angles, represented with their hydrophobic properties
wNc(Z1)_NO_UFR_MI50	Topology of **unfolding-promoters** represented by their hydrophobic properties
wNLC(IP)_NO_UFR_MI50	Topology of **unfolding-promoters** represented with their isoelectric points
wCTP(IP)_NO_GLY_G	Topology of **Gly** residues represented with the isoelectric point
wNc(IP)_NO_GLY_N2	Topology of **Gly** residues represented with the isoelectric point
A_NO_RTR_CV	Area of **residues favoring reverse turns**
wFLC(IP)_NO_RTR_DE	Topology of **residues favoring reverse turns** represented with their isoelectric points
wPsiH(ISA)_NO_RTR_N1	**Residues favoring reverse turns**, with α-helix-like *phi* angles, represented with their non-polar area
wNc(IP)_NO_RTR_P2	Topology of **residues favoring reverse turns** represented with their isoelectric points
ΔGw_NO_PLR_MI50	Free energy of water molecules coordinated to **polar residues**
wCTP(Z1)_NO_PLR_N1	Topology of **polar residues** represented by their hydrophobic properties
wCTP(ISA)_NO_PCR_Ar	Topology of **positive-charged residues** represented with their non-polar areas
wCTP(IP)_NO_ARG_Q2	Topology of **Arg** residues represented with the isoelectric point.

Table SI-13. Detailed information about the benchmark annotation dataset and the prediction performed for each method/protein. Misclassified cases are highlighted in red font

Locus Id	Function	Benchmark Annotation	EzyPred	EnzymeDetector	ProtDCal
SO_0342	prpF protein required for repair/synthesis of Fe-S center of AcnD *PubMed ID: 14702315*	Non-Enz	Non-Enz	Non-detected	Enz
SO_0506	3-octaprenyl-4-hydroxybenzoate Decarboxylase UbiD *PubMed ID: 782527*	Enz	Non-Enz	**Enz** class (4.1.1.-)	Enz
SO_0887	Peptidylarginine deiminase *PubMed ID: 10377098*	Enz	**Enz** (3.5.-.-)	**Enz** class (3.5.3.12)	Enz
SO_1313	Anhydro-N-acetylmuramic acid kinase *PubMed ID: 15901686*	Enz	Non-Enz	**Enz** class (2.7.1.170)	Enz
SO_1523	NAD kinase *PubMed ID: 11488932*	Enz	**Enz** (2.7.-.-)	**Enz** class (2.7.1.23)	Enz
SO_1597	Omega-3 polyunsaturated fatty acid synthase PfaD subunit *PubMed ID: 12055309*	Enz	Non-Enz	**Enz** class (1.3.1.9)	Enz
SO_1608	7-cyano-7-eazaguanine reductase *PubMed ID: 15767583*	Enz	**Enz** (3.5.-.-)	**Enz** class (1.7.1.13)	Enz
SO_1789	UDP-2,3-diacylglucosamine hydrolase *PubMed ID: 12000770*	Enz	Non-Enz	**Enz** class (3.6.1.54)	Enz
SO_1851	Methyltransferase *PubMed ID: 17010378*	Enz	Non-Enz	**Enz** class (2.1.1.173)	Enz
SO_1963	Homogenetisate 1,2-dioxygenase *PubMed ID: 10876237*	Enz	Non-Enz	**Enz** class (1.13.11.5)	Enz
SO_2042	Sulfite oxidase subunit YedY *PubMed ID: 15355966*	Enz	**Enz** (1.7.-.-)	**Enz** class (1.8.-.-)	Enz
SO_2043	Sulfite oxidase subunit yedZ *PubMed ID: 16042411*	Non-Enz	Non-Enz	Non-detected	Enz
SO_2593	NAD-specific glutamate dehydrogenase *PubMed ID: 10924516*	Enz	Non-Enz	**Enz** class (1.4.1.2)	Enz
SO_2603	Methionine-®-sulfoxide reductase *PubMed ID: 17535911*	Enz	Non-Enz	**Enz** class (1.8.4.14)	Enz
SO_2614	Aminodeoxychorismate lyase *PubMed ID: 11011151*	Enz	Non-Enz	Non-detected	Enz
SO_2627	ATP-dependent Clp protease adaptor protein ClpS *PubMed ID: 11931773*	Non-Enz	Non-Enz	Non-detected	Non-Enz

SO_3014	Chromosome segregation and condensation protein B *PubMed ID: 12100548*	Non-Enz	Non-Enz	Non-detected	Enz
SO_3015	Chromosome segregation and condensation protein A *PubMed ID: 12100548*	Non-Enz	Non-Enz	Non-detected	Enz
SO_3367	tRNA guanine-N(7)-methyltransferase *PubMed ID: 12730187*	Enz	**Enz** (2.1.-.-)	**Enz** class (2.1.1.33)	Enz
SO_3436	tRNA pseudouridine synthase TruD *PubMed ID: 12756329*	Enz	Non-Enz	**Enz** class (5.4.99.27)	Enz
SO_3542	Phosphoketolase *PubMed ID: 16086247*	Enz	Enz (4.9.-.-)	**Enz** class (4.1.2.9)	Enz
SO_3578	Multicopper polyphenol oxidase (laccase) *PubMed ID: 16740638*	Enz	Non-Enz	Non-detected	Enz
SO_3667	Heme iron utilization protein HugZ *PubMed ID: 16376031*	Non-Enz	Non-Enz	Non-detected	Enz
SO_3668	Heme iron utilization protein HugX *PubMed ID: 16376031*	Non-Enz	Non-Enz	Non-detected	Enz
SO_3957	3-deoxy-D-manno-octulosonate 8-phosphate phosphatase *PubMed ID: 12639950*	Enz	Enz (3.6.-.-)	**Enz** class (3.1.3.45)	Enz
SO_4227	S-adenosylmethionine-dependent methyltransferase MraW *PubMed ID: 10572301*	Enz	Non-Enz	**Enz** class (2.1.1.199)	Enz
SO_4398	D-tyrosyl-tRNA deacylase *PubMed ID: 10383414*	Enz	Non-Enz	**Enz** class (3.1.-.-)	Non-Enz
SO_4413	Kynureninase *PubMed ID: 9264543*	Enz	Enz (2.8.-.-)	**Enz** class (3.7.1.3)	Enz
SO_4677	3-deoxy-D-manno-octulosonic-acid kinase *PubMed ID: 10531340*	Enz	Non-Enz	**Enz** class (2.7.1.-)	Enz
SO_4680	CDP-glycerol poly(glycerophosphate) Glycerophosphotransferase *PubMed ID: 10648531*	Enz	Enz (2.7.-.-)	Non-detected	Enz

References

1. Kamide, K. and Dobashi, T. (2000), *Physical Chemistry of Polymer Solutions. Theoretical Background*. Elsevier Science.
2. Ruiz-Blanco, Y.B., García, Y., Sotomayor-Torres, C.M. and Marrero-Ponce, Y. (2010) New Set of 2D/3D Thermodynamic Indices for Proteins. A Formalism Based on "Molten Globule" Theory. *Physics Procedia*, **8**, 63-72.

3. Ruiz-Blanco, Y.B., Marrero-Ponce, Y., Paz, W., García, Y. and Salgado, J. (2013) Global Stability of Protein Folding from an Empirical Free Energy Function. *Journal of Theoretical Biology*, **321**, 44-53.
4. Jiang, L., Kuhlman, B., Kortemme, T. and Baker, D. (2005) A “solvated rotamer” approach to modeling water-mediated hydrogen bonds at protein–protein interfaces. *PROTEINS: Structure, Function, and Bioinformatics*, **58**, 893–904.
5. Kyte, J. and Doolitle, R.F. (1982) A Simple Method for Displaying the Hydropathic Character of a Protein. . *J. Mol. Biol.*, **157**, 105-132.
6. Collantes, E.R. and Dunn-III, W.J. (1995) Amino acid side chain descriptors for quantitative structure-activity relationship studies of peptide analogues. *J. Med. Chem.*, **38**, 2705-2713.
7. Plaxco, K.W., Simons, K.T. and Baker, D. (1998) Contact Order, Transition State Placement and the Refolding Rates of Single Domain Proteins. *J. Mol. Biol.*, **277**, 985-994.
8. Burrage, J.S.a.K. (2006) Predicting residue-wise contact orders in proteins by support vector regression. *BMC Bioinformatics*, **425**.
9. Nolting, B., Schalike, W., Hampel, P., Grundig, F., Gantert, S., Sips, N., Bandlow, W. and Qi, P.X. (2003) Structural determinants of the rate of protein folding. *J. Theor. Biol.* , **223**, 299–307.
10. Micheletti, C. (2003) Prediction of Folding Rates and Transition-State Placement From Native-State Geometry. *PROTEINS: Structure, Function, and Genetics*, **51**, 74–84.
11. Zhou, H. and Zhou, Y. (2002) Folding Rate Prediction Using Total Contact Distance. *Biophysical Journal*, **82**, 458–463.
12. Lehninger. (2005), *Biochemistry*, pp. 76-115.
13. Zamyatin, A.A. (1972) Protein Volume in Solution. *Prog. Biophys. Mol. Biol.* , **24**, 107-123.
14. Hellberg S., S., M., Skagerberg B., Wold, S. (1987) Peptide Quantitative Structure-Activity Relationship, a Multivariate Approach. *J. Med. Chem*, **30**, 1126-1135. .
15. Levitt, M. (1978) Conformational Preferences of Amino Acids in Globular Proteins. *Biochemistry*, **17**.
