# Supplementary material for: Exploring general-purpose protein features for distinguishing enzymes and non-enzymes within the twilight zone
Source: BMC Bioinformatics. 2017 Jul 21;18:349. doi: 10.1186/s12859-017-1758-x (PMC5521120; doi:10.1186/s12859-017-1758-x)
Supplement: Supplementary file 3 — Experiments leading to the selection of the SVM and kernel parameters. (PDF 310 kb) [file 12859_2017_1758_MOESM3_ESM.pdf]

Additional File-3: Experiments leading to parameter and kernel selection for the SVMs

Selection of kernel function

In the next table we present the performance of models built with different kernels using default parameters in Weka 3.7.11. The performance summarized in the table concludes that there is not a significant preference for any specific kernel type.

	Linear	Poly-K (e=2)	Poly-K (e=3)	PUK	RBF
**3D data**	81.58	81.07	81.58	81.92	80.73
**1D data**	77.33	78.10	78.18	78.95	77.59

We definitively decide to use the Pearson VII Function Universal Kernel (PUK), because of the proven higher mapping power of this kernel related to more standard choices like Polykernel or RBF. Baydens *et al*. (2006) discussed precisely the suitability of this kernel when one does not have *a priori* knowledge of the nature of the data. These authors claim that the PUK provides a more generalized approach than other kernels [1]. The PUK function has also been applied successfully to model other protein-related problems [2-5]

Selection of SVM parameters

The penalty parameter (C) of the support vector machine as well as the sigma and omega parameters that controls the PUK function, were systematically explored using a grid search within the following ranges: C, is explored from 2^{-5} to 2^{7} by linearly increasing the exponent value. The sigma (σ) value, which controls the half-width of the peak of the kernel function, was varied from 1 to 21 in steps of one unit. Similarly, the omega (Ω) parameters, which tunes the tailing of the function, was varied from 1 to 210 in intervals of ten units.

Next we present surface plots for the exploration of the 1D and 3D families of features which are the ones that lead to the best final models.

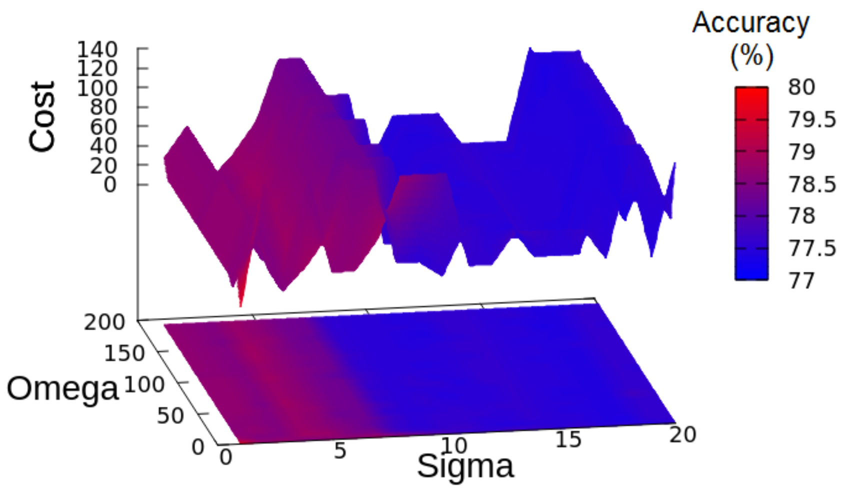

Figure SI3-1. Surface plot of the performance in 5-fold cross validation during the grid search for tuning the parameters of the kernel and the SVM using the 1D descriptor

feature set. The surface represents the value of the cost parameter (C) that maximizes the accuracy for each pair of value of σ and Ω.

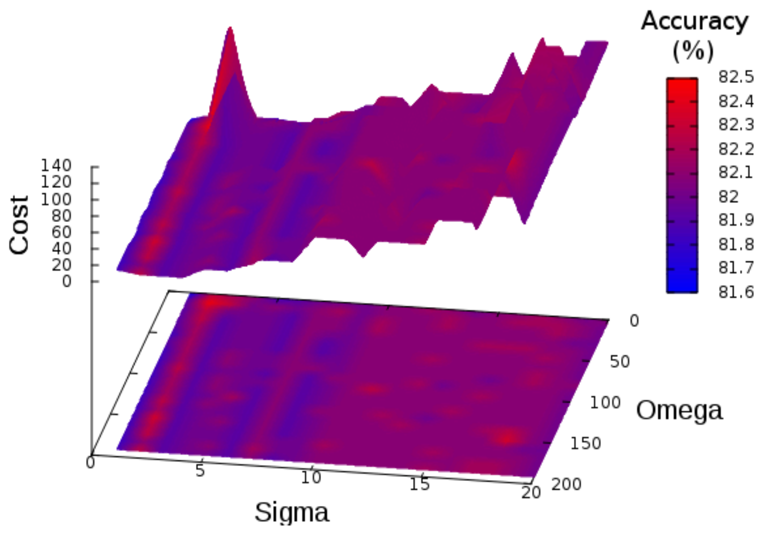

Figure SI3-2. Surface plot of the performance in 5-fold cross validation during the grid search for tuning the parameters of the SVM and kernel using the dataset of 3D descriptors. The surface represents the value of the cost parameter (C) that maximizes the accuracy for each pair of σ and Ω values.

Both explorations show that the classification performance of the models is relatively invariant to the SVM and kernel parameters. In the case of the 3D descriptors, the cost values of the best models tend to increase together with the σ value of the kernel. When using the 1D descriptor data, models with low sigma value show higher accuracy levels, while Ω has no discernable effect. Given the known tendency of SVM classifiers towards over-fitting when using high cost values, and due to the higher observed performance using the 1D data, our attention is focused on favoured region with low σ values. After a detailed analysis of the numeric performance, for each combination of value in the omega-sigma space, the combination (Ω = 1, σ = 1) is selected for the 1D model and (Ω = 11, σ = 2) is selected for the 3D model.

Given the surface plot only shows only one value of the penalty parameter for every combination of omega and sigma, the curves of the performance of the models along different values of penalty (C) are represented in the next figures. The values of Ω and σ are fixed at the above mentioned combinations.

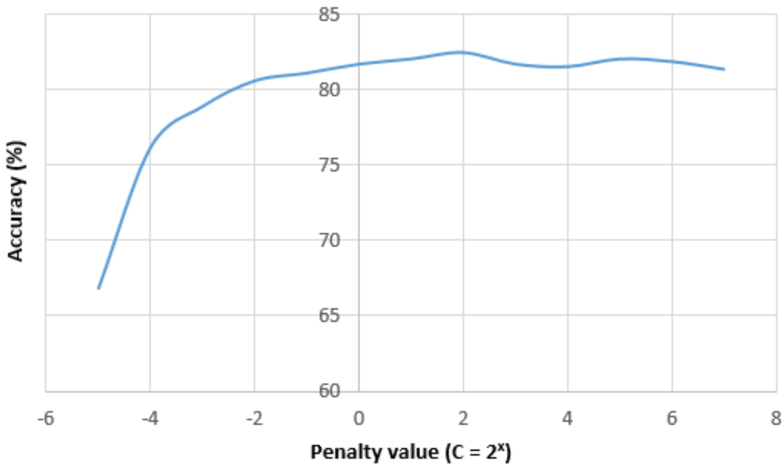

Figure SI3-3. Accuracy in 5-fold cross validation of models built with different values of the penalty parameter (C) using the dataset of 1D descriptors. The values of Ω and σ in the PUK function are fixed at value 11 and 2 respectively.

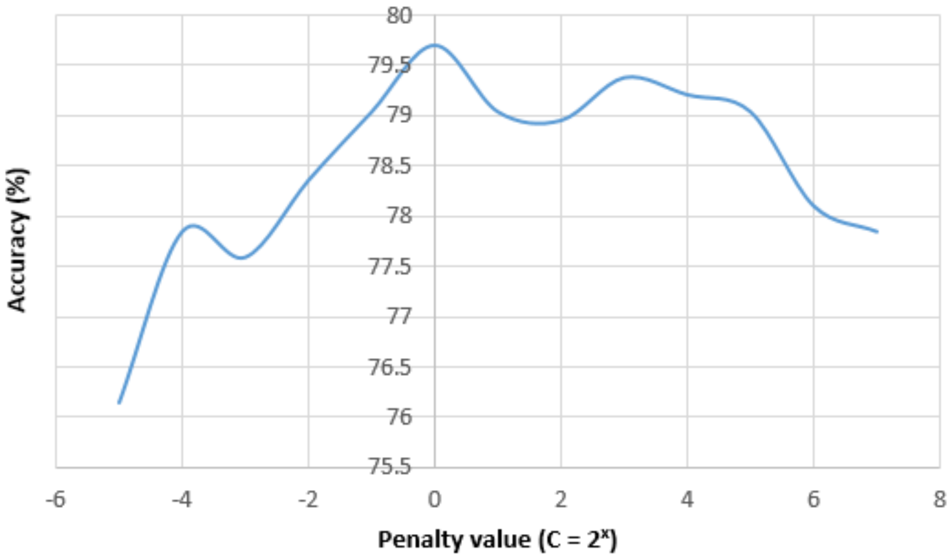

Figure SI3-4. Accuracy in 5-fold cross validation of models built with different values of the penalty parameter (C) using the dataset of 1D descriptors. The values of Ω and σ in the PUK function are both fixed at value 1.

Finally, for the 1D descriptor data, a value a C = 0.5 is selected for the final model, which a value a C = 2 is used for the 3D descriptors.

References

1. Üstün B, Melssen WJ, Buydens LM: **Facilitating the application of Support Vector Regression by using a universal Pearson VII function based kernel**. *Chemometrics and Intelligent Laboratory Systems* 2006, **81**(1):29-40.
2. Zhang G, Ge H: **Support vector machine with a Pearson VII function kernel for discriminating halophilic and non-halophilic proteins**. *Computational biology and chemistry* 2013, **46**:16-22.
3. Qifu Z, Haifeng H, Youzheng Z, Guodong S: **Support vector machine based on universal kernel function and its application in quantitative structure-toxicity relationship model**. In: *Information Technology and Applications, 2009 IFITA'09 International Forum on: 2009*. IEEE: 708-711.
4. Qureshi A, Kaur G, Kumar M: **AVCpred: an integrated web server for prediction and design of antiviral compounds**. *Chemical biology & drug design* 2017, **89**(1):74-83.
5. Sanders WS, Johnston CI, Bridges SM, Burgess SC, Willeford KO: **Prediction of cell penetrating peptides by support vector machines**. *PLoS Comput Biol* 2011, **7**(7):e1002101.
